# The MADS-box genes *SOC1* and *AGL24* antagonize *XAL2* functions in *Arabidopsis thaliana* root development

**DOI:** 10.3389/fpls.2024.1331269

**Published:** 2024-03-21

**Authors:** Claudio A. Castañón-Suárez, Maite Arrizubieta, Natalia Castelán-Muñoz, Diana Belén Sánchez-Rodríguez, Carolina Caballero-Cordero, Estephania Zluhan-Martínez, Sandra C. Patiño-Olvera, J.Arturo Arciniega-González, Berenice García-Ponce, María de la Paz Sánchez, Elena R. Álvarez-Buylla, Adriana Garay-Arroyo

**Affiliations:** ^1^ Laboratorio de Genética Molecular, Epigenética, Desarrollo y Evolución de Plantas, Instituto de Ecología, Universidad Nacional Autónoma de México, Ciudad de México, Mexico; ^2^ Posgrado en Ciencias Biológicas, Universidad Nacional Autónoma de México, Mexico City, Mexico; ^3^ Postgrado en Recursos Genéticos y Productividad-Fisiología Vegetal, Colegio de Postgraduados, Texcoco, Estado de México, Mexico; ^4^ Centro de Ciencias de la Complejidad (C3), Universidad Nacional Autónoma de México, Ciudad de México, Mexico

**Keywords:** MADS-domain proteins, root growth, primary root development, stem cell niche, columella stem cell differentiation, cell wall, quiescent center identity

## Abstract

MADS-domain transcription factors play pivotal roles in numerous developmental processes in *Arabidopsis thaliana.* While their involvement in flowering transition and floral development has been extensively examined, their functions in root development remain relatively unexplored. Here, we explored the function and genetic interaction of three MADS-box genes (*XAL2*, *SOC1* and *AGL24*) in primary root development. By analyzing loss-of-function and overexpression lines, we found that *SOC1* and *AGL24*, both critical components in flowering transition, redundantly act as repressors of primary root growth as the loss of function of either *SOC1* or *AGL24* partially recovers the primary root growth, meristem cell number, cell production rate, and the length of fully elongated cells of the short-root mutant *xal2-2*. Furthermore, we observed that the simultaneous overexpression of *AGL24* and *SOC1* leads to short-root phenotypes, affecting meristem cell number and fully elongated cell size, whereas *SOC1* overexpression is sufficient to affect columella stem cell differentiation. Additionally, qPCR analyses revealed that these genes exhibit distinct modes of transcriptional regulation in roots compared to what has been previously reported for aerial tissues. We identified 100 differentially expressed genes in *xal2-2* roots by RNA-seq. Moreover, our findings revealed that the expression of certain genes involved in cell differentiation, as well as stress responses, which are either upregulated or downregulated in the *xal2-2* mutant, reverted to WT levels in the absence of *SOC1* or *AGL24*.

## 1 Introduction

The MADS-domain family of transcription factors (TFs) is involved in different developmental processes in fungi, plants, and animals (Jamai et al., 2002; Smaczniak et al., 2012a; Cao et al., 2016). In plants, in contrast with animals, this gene family has undergone multiple duplications, resulting in a large family of TFs that participate in many stages of *Arabidopsis thaliana* (from now on Arabidopsis) development (Smaczniak et al., 2012a). Phylogenetic analyses have classified MADS-box genes into two types: type I, or SRF-like genes, and type II MIKC or MEF2-like genes (Alvarez-Buylla et al., 2000; De Bodt et al., 2003; Gramzow et al., 2010). Plant MIKC TFs have four domains; M for MADS, I for intervening sequence, K for keratin-like, and C for C-terminus (Ma et al., 1991). The MADS domain binds to the DNA in the so-called CArG-boxes, with the consensus sequence: [5’-CC(A/T)_6_GG-3’] and its variants (Kaufmann et al., 2005; Zobell et al., 2010). These TFs bind to DNA as homo- or hetero-dimers and exert their regulatory function as tetrameric protein complexes (Schwarz-Sommer et al., 1992; Goto and Meyerowitz, 1994; Davies et al., 1996; Pelaz et al., 2000; Honma and Goto, 2001; Theißen and Saedler, 2001; Sridhar et al., 2006; Brambilla et al., 2007; Immink et al., 2009; Melzer and Theißen, 2009; Smaczniak et al., 2012b).

Historically, MADS-box genes have primarily been investigated in the context of flowering transition, vernalization and the determination of floral organ identity, which has led to the establishment of a combinatorial “ABC model” of flower development (Coen and Meyerowitz, 1991; Borner et al., 2000; Lee et al., 2000; Honma and Goto, 2001; Theißen and Saedler, 2001; Moon et al., 2003; Searle et al., 2006; Gramzow et al., 2010; Lee and Lee, 2010). The ABC model explains how combinatorial interactions among MADS-domain proteins expressed in specific parts of the floral meristem, specify the whorls where floral organs will develop (Theißen and Saedler, 2001; Melzer and Theißen, 2009).

It has been shown that there are over 45 genes that participate in the Gene Regulatory Network (GRN) involved in flowering transition (Chávez-Hernández et al., 2022) including *SOC1* (*SUPPRESSOR OF OVEREXPRESSION OF CONSTANS 1*), *AGL24* (*AGAMOUS-LIKE 24*) and *XAL2/AGL14* (*XAANTAL2/AGAMOUS-LIKE 14*). These three genes have been described as promoters of flowering transition, as their loss-of-function mutants show a late-flowering phenotype under both long- and short-day conditions, whereas the overexpression lines display early flowering phenotypes (Borner et al., 2000; Lee et al., 2000; Samach et al., 2000; Hepworth et al., 2002; Moon et al., 2003; Lee and Lee, 2010; Pérez-Ruiz et al., 2015). Interestingly, the proteins encoded by these MADS-box genes interact with each other in a yeast two-hybrid system (De Folter et al., 2005). Additionally, AGL24 and SOC1 exhibit redundant roles in flowering transition, and the formation of the SOC1-AGL24 heterodimer is crucial for SOC1 nuclear localization (Lee et al., 2008). Furthermore, there is a mutual upregulation between *SOC1* and *AGL24* (Liu et al., 2008), while another MADS-box gene, *XAL2*, which is also described as a promoter of primary root development (Garay-Arroyo et al., 2013), enhances and represses *SOC1* and *AGL24* in the shoot (Pérez-Ruiz et al., 2015).

The expression of the *AGL24* and *SOC1* MADS-box genes in roots (Lee et al., 2000; Michaels et al., 2003) indicates their potential roles in this tissue, consistent with the previously reported function of *XAL2* (Garay-Arroyo et al., 2013). The Arabidopsis primary root has emerged as a valuable model for studying the interplay between proliferation and differentiation rates, which collectively establish the morphogenetic pattern. In the Arabidopsis primary root, three zones with different cellular behaviors can be distinguished: the Root Apical Meristem (RAM) that contains the Stem Cell Niche (SCN), the proliferation (PD) and the transition domains (TD), the elongation zone (EZ) and the maturation zone (MZ). These zones are found along the longitudinal axis from the root tip to the hypocotyl, and all the postembryonic cells are derived from the SCN containing an organizer center known as the Quiescent Center (QC). The QC is surrounded by five sets of initial cells (Dolan et al., 1993; Scheres et al., 2002; Di Mambro et al., 2019) that remain in an undifferentiated state (Van den Berg et al., 1997). Following their passage through the SCN, cells undergo approximately 4-6 rounds of proliferation within the RAM. Subsequently, they transit into the anisotropic and rapidly elongating Elongation Zone (EZ), where cells enlarge at a high rate. Afterwards they acquire their definitive characteristics within the MZ (Baluska and Volkmann, 2001; Baluska et al., 2010; Ivanov and Dubrovsky, 2013). Towards the root tip and distally to the QC, the Columella Stem Cells (CSC) undergo division to give rise to the Differentiated Columella Cells (DCC).

In this work, we explored the roles of *XAL2* along with the participation of *SOC1* and *AGL24* in primary root development using single, double, and triple loss of function mutants of these genes, as well as overexpression lines. Our study revealed that *SOC1* and *AGL24* antagonize many *XAL2* functions, as their absence partially recovered the root and cellular phenotypes of the loss-of-function mutant *xal2-2*, as observed in the double mutants *xal2-2 agl24-4* and *xal2-2 soc1-6*. In addition, SOC1 was identified as a negative regulator of columella stem cell differentiation. Also, we found that the transcript levels of *PI4KG3*, a salt and osmotic stress-responsive gene, are higher in *xal2-2*, suggesting the absence of *XAL2* is sufficient to trigger a salt and osmotic stress response. Interestingly, the altered *PI4KG3* expression levels return to WT levels in the double mutants *xal2-2 agl24-4* and *xal2-2 soc1-6*. Collectively, our data suggest the involvement of these three MADS-box genes alone or in combination in the primary root development.

## 2 Results

### 2.1 *XAL2, SOC1* and *AGL24* transcripts and their proteins are accumulated in root tissues

**Figure 1:**
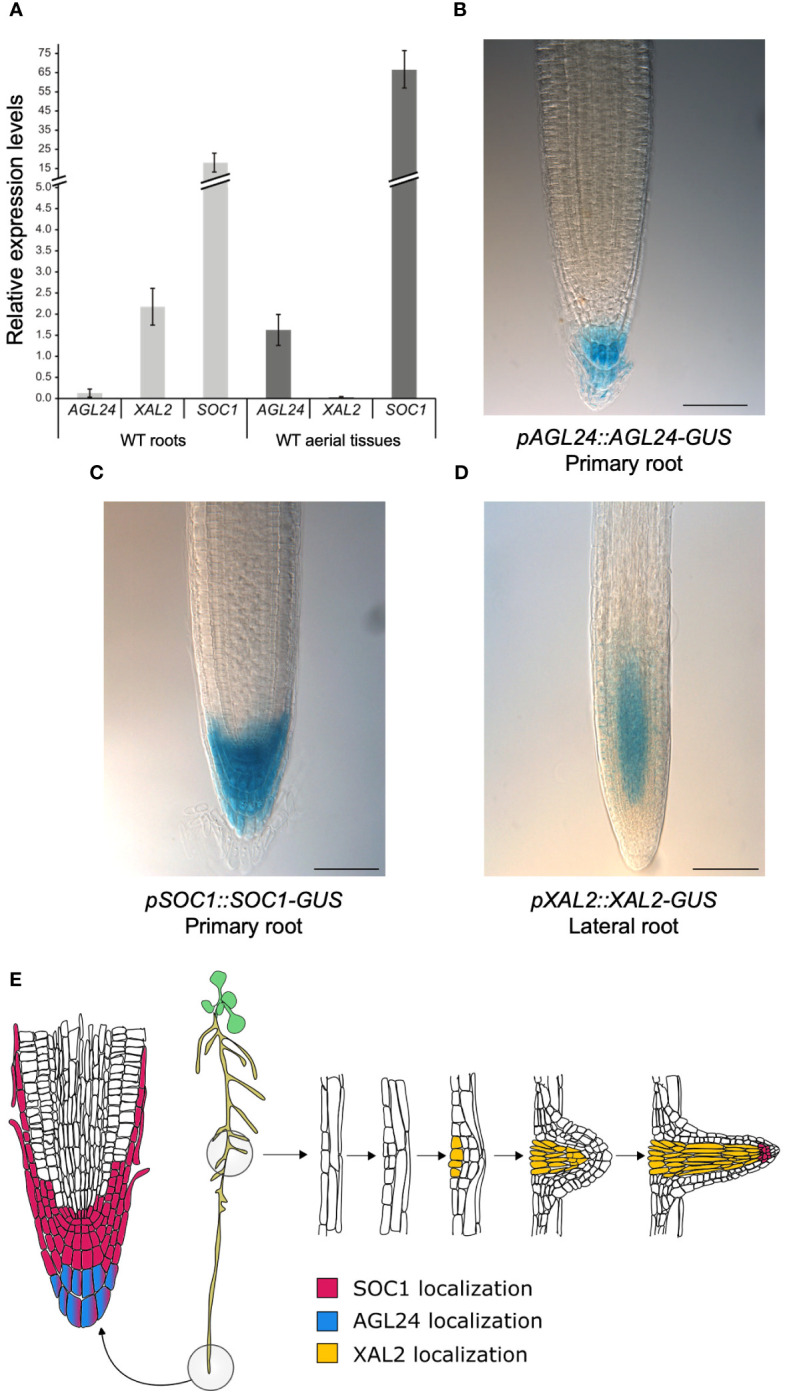
*AGL24*, *XAL2* and *SOC1* gene expression and protein accumulation patterns in root tissues. **(A)** Expression analysis by RT-qPCR of *AGL24*, *SOC1* and *XAL2* in roots and aerial tissues (including hypocotyls, cotyledons, leaves, and SAM) of 7 dps WT plants. Expression levels are relative to *RNAH, PDF2* and *UPL7* levels in WT roots and aerial tissues. Data is presented as the mean ± SD of three independent biological replicates with two technical replicates each. Root tissue localization of AGL24 **(B)**, SOC1 **(C)** and XAL2 **(D)** proteins. Histochemical GUS staining of 7 day-post-sowing (dps) Arabidopsis primary roots carrying the *pAGL24::AGL24-GUS* and *pSOC1::SOC1-GUS* constructs and lateral roots of 7 dps plants carrying the *pXAL2::XAL2-GUS* construct. Scale bar = 75 μM. Representative root pictures are presented, *n*= 30. **(E)** Schematic representation illustrating the spatial localization of SOC1, AGL24, and XAL2 proteins in Arabidopsis primary and lateral root tissues. Based on Encyclopédie de l'invironment (2024).

*XAL2*, *SOC1* and *AGL24* are important components of the GRN governing the flowering transition (Borner et al., 2000; Yu et al., 2002; Pérez-Ruiz et al., 2015) and protein-protein interactions for these TFs have been demonstrated by *in vitro* yeast-two-hybrid experiments (De Folter et al., 2005). Since *XAL2* is a promoter of Arabidopsis primary root growth (Garay-Arroyo et al., 2013), we decided to further investigate its role in root development in combination with *SOC1* and *AGL24*. To establish if *SOC1* and *AGL24* participate in primary root growth, we first analyzed their gene expression in both roots and aerial tissues by comparing their expression levels with those of *RNAH*, *UPL7* and *PDF2* housekeeping genes (Czechowski et al., 2005; Hong et al., 2010). In 7 day-post-sowing (dps) plants, we found that *SOC1* is highly expressed in both roots and aerial tissues, whereas *AGL24* expression levels are intermediate in aerial tissues, but extremely low in roots (**Figure 1A**). In contrast, *XAL2* shows intermediate expression levels in roots, while showing minimal expression levels in aerial tissues (**Figure 1A**).

Moreover, we analyzed the expression patterns of these genes using transcriptional promoter-GUS/GFP fusions (**Supplementary Figures 1A**, **2A**, **3A**). We found that *XAL2* is mainly expressed in the proximal vascular bundle of the primary root, near the hypocotyl (**Supplementary Figure 1B**), as previously reported (Garay-Arroyo et al., 2013). Despite previous *in situ* hybridization analyses suggesting the presence of *XAL2* transcripts in the primary root meristem (Garay-Arroyo et al., 2013), we did not observe *GUS* expression driven by a 1127 bp *XAL2* promoter in this region (**Supplementary Figure 1B**). *SOC1* is highly expressed in the primary root tip, specifically in the stem cell niche, lateral root cap and columella cell layers (**Supplementary Figure 2B**) as well as in lateral root meristems, the proximal primary root and the hypocotyl (**Supplementary Figure 2C**), as in previous reports (Hepworth et al., 2002). *AGL24* was found to be highly expressed in the vascular bundles of roots and leaves and in the shoot apical meristem (SAM) (**Supplementary Figure 3B**), as previously reported (Huang et al., 2024). Furthermore, we also analyzed the protein accumulation pattern of SOC1, AGL24, and XAL2 in root tissues. It has been demonstrated that the intronic regions in the MADS-box genes play important roles for their regulation (Sieburth and Meyerowitz, 1997; Deyholos and Sieburth, 2000; Kooiker et al., 2005; De Folter et al., 2007; Schauer et al., 2009; Singer et al., 2010), so we generated genetic constructs using the genomic regions of these MADS-box genes, which included intronic regions and excluded the stop codon. These constructs were fused with the *GUS* reporter gene and placed under the control of their respective native promoters (refer to **Supplementary Figures 1A**, **2A**, **3A** for a schematic representation).

We analyzed the protein accumulation patterns of AGL24, SOC1 and XAL2 in 7 dps transgenic lines grown in MS plates. We observed that AGL24 is only accumulated in the differentiated columella cell layers of primary roots (**Figures 1B, E**, **Supplementary Figure 3D**) and no AGL24-GUS signal was detected in lateral root primordia nor in lateral roots (**Supplementary Figure 3C**). We found a strong AGL24-GUS signal in the SAM (**Supplementary Figure 3C**), which is a similar pattern to the previously reported by *in situ* hybridization assays (Michaels et al., 2003). In contrast with the *pAGL24::GUS* transcriptional pattern (**Supplementary Figure 3B**), when analyzing the protein localization pattern, no AGL24-GUS signal was detected in the vascular tissues of roots or shoots or in lateral root meristem (**Supplementary Figure 3C**).

In addition, similarly to its expression pattern, the SOC1 protein accumulates in the proximal zone of the RAM, specifically in the stem cell niche, lateral root cap and columella cell layers (**Figures 1C, E**). While no SOC1-GUS signal was detected in lateral root primordia, it was observed in later stages of lateral root development (**Supplementary Figure 2D**). Additionally, the SOC1 protein is present in axillary buds and cotyledons, but it is absent in the SAM and in the primary root zone close to the hypocotyl at this developmental stage, which contrasts with the expression pattern observed with the promoter-GUS fusion (**Supplementary Figure 2C, D**).

We found that the XAL2 protein is mainly localized in lateral root meristems of 7 dps plants, specifically in the vascular cylinder of the meristematic zone of lateral roots, even in early stages of development, as well as in lateral root primordia and lateral root vascular tissues (**Figures 1D, E**, **Supplementary Figures 1C**, **4F-G**). Despite the *XAL2* transcript being present in the primary root (**Supplementary Figure 1B**; Garay-Arroyo et al., 2013), we could not detect its protein in this tissue using our constructs (**Supplementary Figures 1C**, **4E**). However, the short-root phenotype of the *xal2-2* mutant is reverted when this mutant is transformed with a *pXAL2::XAL2-GFP* construct (**Supplementary Figures 4A–D**), whereas the overexpression line *35S::GFP-XAL2* displayed a longer root phenotype (**Supplementary Figures 4C, D**). Additionally, the XAL2 protein was observed in the trichomes of leaves during early developmental stages (**Supplementary Figure 1D**).

We described that AGL24 and SOC1 are present in root tissues where they could potentially interact to form dimers in the columella differentiated cells (**Figure 1E**). Furthermore, it seems unlikely that XAL2 could interact with AGL24 in roots, as their localization patterns in this organ do not overlap (**Figure 1E**).

In summary, these results indicate that the *SOC1* and *AGL24* transcripts and their proteins, which are integral components of the flowering transition GRN, are present in root tissues and may play a role in primary root growth.

### 2.2 *SOC1* and *AGL24* redundantly repress primary root growth

**Figure 2:**
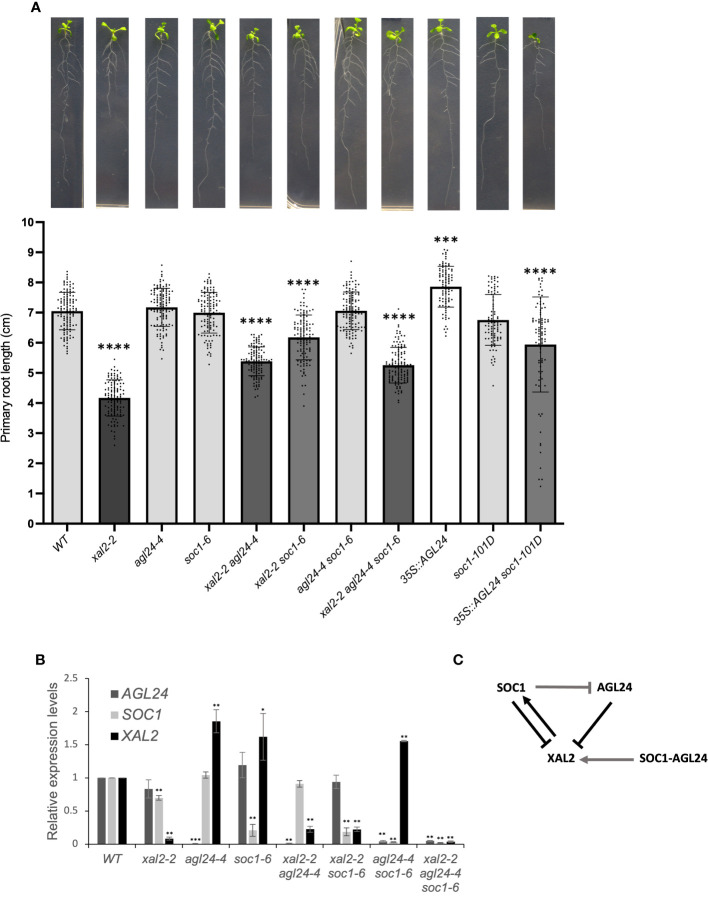
*SOC1* and *AGL24* are repressors of primary root growth. **(A)** Primary root length (cm) of 7 day-post-transplantation (dpt) plants, including WT, *xal2-2*, *agl24-4*, *soc1-6*, the double mutants *xal2-2 agl24-4*, *xal2-2 soc1-6*, *agl24-4 soc1-6*, the triple mutant *xal2-2 agl24-4 soc1-6*, the single overexpression lines *35S::AGL24*, *soc1-101D*, and the double overexpression line *35S::AGL24 soc1-101D* (n ≥ 85 plants). Data is presented as the mean ± SD (n ≥ 85). Asterisks denote significant differences compared to WT roots and different shades of gray in the bars indicate significant differences among lines (Kruskal-Wallis test followed by Dunn’s *post-hoc* test, *** P<0.002; **** P< 0.0001). **(B)** Relative expression levels of *AGL24*, *SOC1* and *XAL2* in roots of 7 days post sowing (dps) in the different lines. Data is presented as mean ± SD of three independent biological replicates with two technical replicates. Asterisks indicate significant differences compared to WT plants (Student’s t-test or Wilcoxon Mann-Whitney test, * P<0.05, ** P<0.005, *** P<0.0005). **(C)** Model for the regulation of the expression of *XAL2*, *AGL24* and *SOC1* in Arabidopsis root. Arrows indicate induction and bar-lines repression. Black lines indicate genetic interactions observed in the loss-of-function lines, gray lines indicate interactions observed in the overexpression lines and *SOC1* and *AGL24* together (SOC1-AGL24) mean how the co-overexpression of these genes regulates *XAL2*.

To analyze the *SOC1* and *AGL24* function in roots, we used two loss of function mutant alleles for each gene (*soc1-2* and *soc1-6*, *agl24-3* and *agl24-4*; see **Supplementary Figures 5A, B** for genomic structure and expression levels, respectively) and a double mutant *agl24-4 soc1-6* was generated. These mutants were grown for 12 dps in vertical petri dishes, and the primary root growth was measured for 7 days starting from the day of transplantation (day 5). The primary root growth kinetics of *soc1-6* (and *soc1-2*), *agl24-4* as well as the double mutant *agl24-4 soc1-6* (**Supplementary Figures 6A, C**) showed no significant differences in root length at 12 dps in comparison with WT plants; however, we found that the root length of *agl24-3* is slightly but significantly longer than WT plants since day 3 post transplantation (**Supplementary Figure 6B**). Furthermore, the double overexpression line *35S::AGL24 soc1-101D* showed significantly shorter roots compared to WT plants despite the *35S::AGL24* line showing slightly, but significantly longer roots, and the primary root length observed in *soc1-101D* was not different from WT plants (**Figures 2A**, **Supplementary Figure 6D**).

In addition, the absence of either *SOC1* or *AGL24* is sufficient to partially recover the primary root length of the short-root mutant *xal2-2* since day 4 post transplantation (**Supplementary Figure 6C**). The root length of *xal2-2 soc1-6* and *xal2-2 agl24-4* double mutants was significantly shorter than WT plants, but longer than *xal2-2* (**Figures 2A**; **Supplementary Figure 6C**; **Supplementary Table 1**). Additionally, the *xal2-2 soc1-6* double mutant exhibited a longer root than that observed in the *xal2-2 agl24-4* double mutant since day 4 post transplantation, indicating that the absence of *SOC1* reversed more significantly the *xal2-2* short-root phenotype. These intermediate phenotypes were observed in two different *SOC1* and *AGL24* mutant alleles (**Supplementary Figures 6A, B**) and indicate that *SOC1* and *AGL24* are root growth repressors in the *xal2-2* loss-of-function mutant and participate in a different pathway from that of *XAL2* regarding primary root growth (**Figures 2A**; **Supplementary Figure 6C**).

Moreover, the primary root length of the triple mutant *xal2-2 agl24-4 soc1-6* is significantly longer than that of the *xal2-2* mutant, but not significantly different from the *xal2-2 agl24-4* double mutant, suggesting that *AGL24* could be epistatic over *SOC1* in primary root growth in this genetic interaction (**Figure 2A**).

All this data suggests that *SOC1* and *AGL24* redundantly counteract the role of *XAL2* in primary root growth, while simultaneously participating in independent pathways, despite these three genes function as promoters of the flowering transition (Pérez-Ruiz et al., 2015).

### 2.3 Gene regulatory interactions among *SOC1*, *AGL24* and *XAL2* in root development

In the SAM, *SOC1* and *AGL24* form a positive feedback regulatory loop (Lee et al., 2008), while *XAL2* exerts both positive and negative regulation on the expression of both *SOC1* and *AGL24* (Pérez-Ruiz et al., 2015). Additionally, the expression of *XAL2* is downregulated by the overexpression of *SOC1*, whereas the absence or overexpression of *AGL24* does not impact the expression of *XAL2* (Pérez-Ruiz et al., 2015). It is important to clarify that, by regulation, we mean that some genes are either down- or upregulated in the different backgrounds compared to the WT, and not that they are directly regulated by that transcription factor. To explore the cross-regulation of these genes in Arabidopsis roots, we analyzed the gene expression in single, double and triple mutant backgrounds. As expected, the expression levels of the three MADS-box genes in their respective loss of function mutants were low in all cases (**Figures 2B**, **Supplementary Figures 5B, C**).

As shown in **Figure 2B**, *AGL24* and *SOC1* are negative regulators of *XAL2* in roots as *XAL2* expression levels are higher in *agl24-4*, *soc1-6* and in the double mutant *agl24-4 soc1-6* when compared to WT levels in roots. A similar tendency was observed with the different alleles *agl24-3*, *soc1-2*, *xal2-2 agl24-3* and *xal2-2 soc1-2* (**Supplementary Figure 5C**). However, *XAL2* expression levels are also significantly higher in the double overexpression line *35S::AGL24 soc1-101D* (**Supplementary Figure 5D**), suggesting a non-linear expression regulation of this gene by AGL24 and SOC1 in conjunction. Consistent with previous findings in aerial tissues (Pérez-Ruiz et al., 2015), XAL2 upregulates *SOC1* expression in roots, as *SOC1* expression levels are significantly lower in *xal2-2.* Interestingly, in contrast to the gene regulation observed in the shoot (Pérez-Ruiz et al., 2015), *XAL2* does not affect *AGL24* expression in the roots, as the *AGL24* expression levels in *xal2-2* are similar to those in WT plants (**Figures 2B–D**).

Moreover, in the overexpression lines (where the relative expression levels of each gene increases considerably, **Supplementary Figure 5D**) the *AGL24* expression in roots is inhibited by the overexpression of *SOC1*, whereas the expression levels of *SOC1* are not affected by the overexpression of *AGL24* (**Supplementary Figure 5D**) differently to what has been shown for the shoot (Michaels et al., 2003; Liu et al., 2008; Pérez-Ruiz et al., 2015).

In summary, the genetic regulation of these genes is different between the shoot and the root. In addition, *SOC1* together with *AGL24* are necessary for maintaining the specific expression levels of *XAL2* in roots, the latter upregulates *SOC1* expression and SOC1 downregulates *AGL24* expression (**Figure 2C**).

### 2.4 The loss of function of *SOC1* and *AGL24* in the primary root compensates for the disrupted cellular homeostasis in *xal2-2* mutants

**Figure 3:**
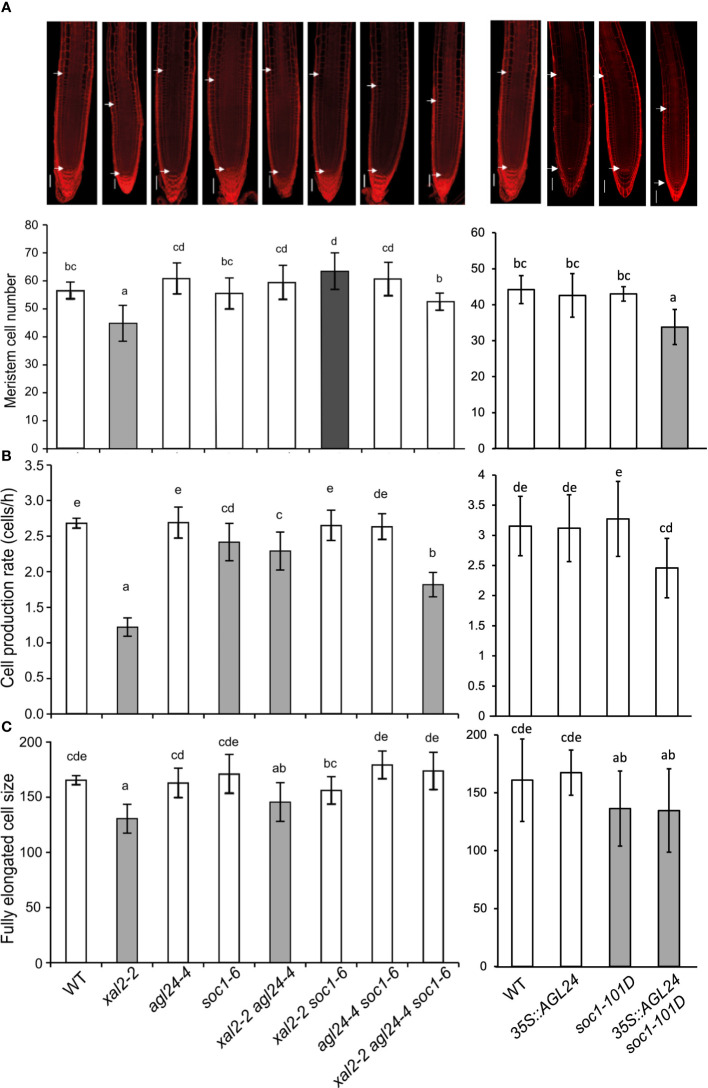
*SOC1* and *AGL24* antagonize the *XAL2* function in meristem cell number, fully elongated cell size and cell production rate of primary root. **(A)** Meristem cell number (Bars, 50 μM); **(B)** cell production rate and **(C)** size of the fully elongated cells of 6 dps seedlings of WT, *xal2-2, agl24-4, soc1-6* the double mutants *xal2-2 agl24-4, xal2-2 soc1-6, agl24-4 soc1-6*, the triple mutant *xal2-2 agl24-4 soc1-6, 35S::AGL24, soc1-101D and* the double overexpression line *35S::AGL24 soc1-101D*. The gray and white bar colors denote significant differences with WT plants. Different letters indicate statistical differences among the different lines (ANOVA followed by a Tukey *post-hoc* test). Data are presented as the mean ± SD of n ≥ 9 plants.

To further understand the cellular basis of the uncovered single and multiple *SOC1*, *AGL24* and *XAL2* mutant root phenotypes, we measured the number of cortex cells in the meristem and the length of fully elongated cells. In general, our results showed that the primary root growth is correlated to meristem cell number (ρ= 0.69 for WT vs loss-of-function mutants and 0.68 for WT vs overexpression lines) and cell production rate (ρ= 0.89 for WT vs loss-of-function mutants and 0.66 for WT vs overexpression lines) and highly correlated with fully elongated cell size (ρ= 0.89 for WT vs loss-of-function mutants and 0.87 for WT vs overexpression lines). As previously reported, the short-root phenotype in *xal2-2* could be explained by a reduction in the number of cells in the meristem, shorter fully elongated cells, and a slower cell production rate (Garay-Arroyo et al., 2013). Interestingly, a similar pattern was observed in the double overexpression line *35S::AGL24 soc1-101D*, which could explain its shorter primary root (**Figure 3**). In addition, *agl24-4* and *soc1-6* had the same meristem cell number and fully elongated cell size as the WT, which correlates with its root growth. The double mutant *xal2-2 agl24-4* exhibits an intermediate cell production rate when compared to the single mutants. Meanwhile, the meristem cell number is similar to that of *agl24-4*, but the fully elongated size resembles that of *xal2-2*. This observation may provide an explanation for its primary root length. In addition, the double mutant *xal2-2 soc1-6* displays the largest meristem cell number, whereas the fully elongated cell size is significantly larger than *xal2-2* but not significantly different from *soc1-6* or the WT (**Figure 3**).

In summary, our quantitative cellular analyses of these mutants suggest that the loss of function of either *AGL24* or *SOC1* is sufficient to partially recover the quantitative cell phenotypes observed in the *XAL2* mutant, whereas their co-overexpression leads to root growth inhibition with similar cell phenotypes as those presented in *xal2-2*.

### 2.5 *SOC1* negatively regulates columella stem cell differentiation

The number of cells in the RAM depends on the number of cells that are produced in the SCN, the proliferation rate of these cells, as well as the cells that transit to the Elongation Zone. As we did not observe evident defects in SCN morphology in *AGL24* and *SOC1* mutants and given that SOC1 is localized in the SCN and columella cells, and AGL24 is only localized in the differentiated columella cells layers (**Figures 1B, C, E**), we analyzed if the distal stem cells in loss- and gain-of-function lines had undergone premature or late differentiation. The columella stem cells (CSCs) can be distinguished from the columella differentiated cells (DCCs) by the staining of starch grains of DCCs with Lugol (Van den Berg et al., 1997). We analyzed 5 dps plants for all the mutant lines and 5 and 6 dps plants for the double mutant line *35S::AGL24 soc1-101D*, as a previous germination assay showed that this line presented a slower germination rate than the rest of the lines (around 12-20 h later compared with WT) (**Supplementary Figure 7**). We decided not to consider the cell layers that are partially detached from the root (for an example, see black arrow in **Supplementary Figure 8** in *agl24-4* representative photographs of the root tip).

**Figure 4:**
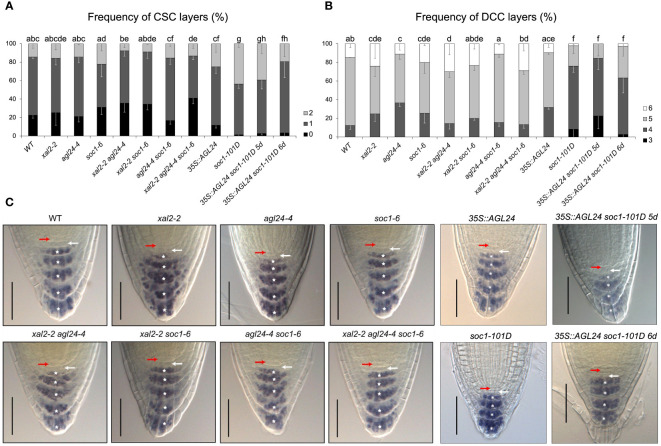
*XAL2*, *SOC1* and *AGL24* regulate columella cell differentiation. Frequency of plants with different numbers of **(A)** columella stem cells layers (CSC) and **(B)** differentiated columella cells layers (DCC) characterized by the accumulation of starch grains. Observations were conducted on 5 dps plants of WT, *xal2-2*, *agl24-4*, *soc1-6*, the double mutants *xal2-2 agl24-4*, *xal2-2 soc1-6*, *agl24-4 soc1-6*, the triple mutant *xal2-2 agl24-4 soc1-6, 35S::AGL24, soc1-101D* and the double overexpression line *35S::AGL24 soc1-101D*. The double overexpression line *35S::AGL24 soc1-101D* was also analyzed at 6 dps. Data is shown as the mean ± SE of three independent biological replicates (n > 85). Letters represent significant differences among lines as determined by a Fisher test followed by Bonferroni correction. **(C)** Representative images of the root tip of the different lines; the red arrow shows the QC, the white arrow the CSC layer and the asterisks indicate the different DCC layers. Bars, 50 μM.

We found significant differences in CSC phenotypes only in the triple mutant *xal2-2 agl24-4 soc1-6*, in *soc1-101D*, and in the double overexpression line (*35S::AGL24 soc1-101D*) at both 5 and 6 days, compared to WT plants (**Figure 4A**). Interestingly, we found that the overexpression of *SOC1* is sufficient to induce a phenotype significantly different from the observed in the rest of the analyzed lines (WT, *xal2-2*, *agl24-4*, *soc1-6*, *xal2-2 agl24-4*, *xal2-2 soc1-6*, *agl24-4 soc1-6*, *xal2-2 agl24-4 soc1-6*, *35S::AGL24*) (**Figure 4A**; **Supplementary Tables 2**, **3**). Specifically, we observed a reduced proportion of plants with no CSC layers in *soc1-101D* (1.2%) and in the double overexpression line *35S::AGL24 soc1-101D* at 5 and 6 dps plants (2.6% and 3.3%, respectively), compared to WT plants (14.5%) (**Figures 4A–C**; **Supplementary Tables 2**, **3**).

In addition, the frequency of DCC layers was significantly different from the WT in single mutant lines (*xal2-2*, *agl24-4* and *soc1-6*). On the other hand, there were no significant changes in the double and triple mutants *xal2-2 soc1-6* and *agl24-4 soc1-6, xal2-2 agl24-4 soc1-6*, as well as in *35S::AGL24* (**Figure 4B**, **Supplementary Tables 2**, **3**). Interestingly, the overexpression of *SOC1* caused a notorious phenotype in terms of DCC number. In 5 dps plants, the lines overexpressing *SOC1* predominantly exhibited only four layers of DCCs (67.7% for *soc1-101D* and 61.8% for *35S::AGL24 soc1-101D*), in contrast to WT plants (12.34%), where most plants had five layers of DCCs. We also analyzed 6 dps *35S::AGL24 soc1-101D* plants and found that most plants (60.5%) still had only 4 DCC layers (**Figures 4B–C**, **Supplementary Tables 2**, **3**). This suggests that the observed phenotype was a result of the overexpression of *SOC1* rather than differences in the developmental stages. Therefore, we propose SOC1 as a clear negative regulator of columella cell differentiation.

### 2.6 RNA-seq analyses revealed global XAL2-regulated genes

**Figure 5:**
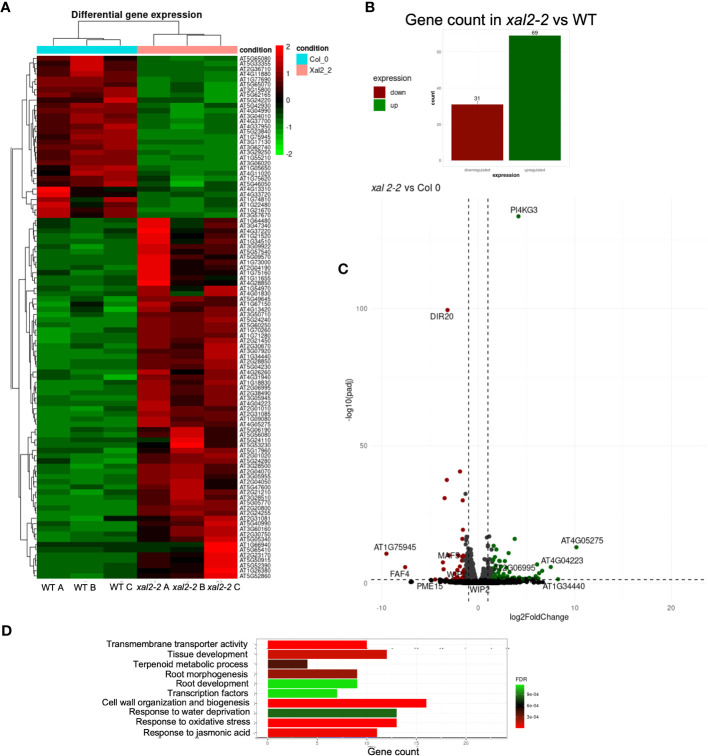
Identification of differentially expressed genes (DEGs) in *XAL2* mutant vs WT plants. **(A)** Heatmap illustrating the expression profiles of all differentially expressed genes (DEGs) identified through RNA-seq analysis of 7 dps wild-type (WT) and *xal2-2* mutant roots. The data encompasses three independent biological replicates for each genetic background, denoted as A, B, and C for clarity. In the heatmap, elevated and reduced expression levels are represented by red and green coloration, respectively. Transitions in color represent changes in gene expression levels. **(B)** The number of DEGs in this comparison. **(C)** Volcano plots and statistical data of DEGs in *xal2-2* vs WT comparison. Red and green spots represent downregulated and upregulated DEGs, while gray and black indicate genes with no significant change in expression levels. **(D)** Top 10 Gene Ontology (GO) terms that are enriched for DEGs found in the *xal2-2* vs WT comparison.

Since *xal2-2* mutants exhibited the most prominent short-root phenotype and limited information was available on genes regulated by *XAL2*, we conducted an RNA-seq analysis to identify differentially expressed genes (DEGs) (fold change > 1.5; P<0.05) (**Figures 5A–C**) in 7 dps roots of the loss-of-function mutant *xal2-2* (Garay-Arroyo et al., 2013) and WT plants.

The comparative analysis between *xal2-2* and WT revealed 100 unique DEGs, and the expression of many of these genes is strongly regulated by *XAL2*, as most genes share a similar level of expression within the same genetic background, with only a few genes showing scattered expression (**Figures 5A, B**). We identified 31 downregulated genes and 69 upregulated genes in *xal2-2* compared to WT (**Figures 5B, C**). A list of the 20 most differentially expressed genes found in *xal2-2* is provided in **Supplementary Table 4**.

Gene Ontology (GO) analysis showed that some of the biological processes enriched in the *xal2-2* vs WT comparison were: “Cell wall organization and biogenesis”, “Response to water deprivation” and “Root development” (**Figure 5D**). Among the enriched categories, the levels of some salt stress response genes, such as *PI4KG3/AtPI4Kγ3/MOP9.5* (Akhter et al., 2016), and *WRKY30* (El-Esawi et al., 2019), were significantly higher in *xal2-2* compared with WT. In an opposite way, some biotic and abiotic responsive genes, such as *DIR20* (Paniagua et al., 2017) and *DEFL207* (Kimura et al., 2023), were significantly downregulated in *xal2-2* (**Supplementary Figure 9B**). Moreover, in *xal2-2* we observed higher expression levels of xyloglucan endotransglucosylases/hydrolases (XTHs) such as *XTH12* and *XTH26* as well as some peroxidases (*PER8/PRX8* and *PER52/PRX52*), two classes of enzymes that play important roles in plant cell wall organization and biogenesis (Ishida and Yokoyama, 2022) (**Supplementary Figure 9A**).

Another group of DEGs identified in *xal2-2* includes TFs, including root cell-type specific TFs like *WOX7*, expressed in cortex and endodermis initial cells as well as in the endodermis (Kong et al., 2016), *AGL42/FYF*, a QC-specific MADS-box gene (Nawy et al., 2005) (**Supplementary Figure 9C**) and *WIP2/NTT*, a zinc finger transcription factor that acts redundantly with *WIP4* and *WIP5*, required for the initiation of the root meristem (Crawford et al., 2015). Additionally, another MADS-box gene, *MAF5*, known to inhibit seedling establishment under salt stress (Perrella et al., 2024), was significantly downregulated in *xal2-2* (**Supplementary Figure 9C**).

Collectively, these results show that in the *xal2-2* mutant, the expression of several genes associated to cell wall organization, abiotic stress responses, and cell identity is affected, which in conjunction, might influence the primary root length. Despite there being an evident misregulation of several genes in the absence of *XAL2*, whether the altered expression of these genes is directly regulated by XAL2 needs to be further confirmed by ChIP-qPCR or ChIP-seq experiments.

### 2.7 XAL2, AGL24 and SOC1 regulate genes involved in QC identity, osmotic stress responses and cell wall organization/expansion

To verify the accuracy of DEGs results, we analyzed the expression levels of some genes in all the lines used in this work using RT-qPCR, including *WOX5* and *AGL42*, two QC-specific TFs, given that the loss of function of *WOX5* affects CSC differentiation (Doerner, 1998; Nawy et al., 2005; Sarkar et al., 2007) and *AGL42* is expressed in roots, but it also participates in regulating flower senescence and abscission, similarly to *XAL2 (* Nawy et al., 2005 *;* Chen et al., 2022 *).* We also analyzed *PI4KG3* expression, a type II phosphoinositide 4-kinase that increases tolerance to high salinity or ABA not only by readjusting the accumulation of reactive oxygen species (ROS) but also by inducing the expression of some stress-responsive genes (Akhter et al., 2016) and *PER8*, a class III peroxidase with high expression levels in roots. *PER8* has been shown to be involved in root growth, as loss-of-function mutants of this gene show longer root phenotypes (Jeong et al., 2022).

**Figure 6:**
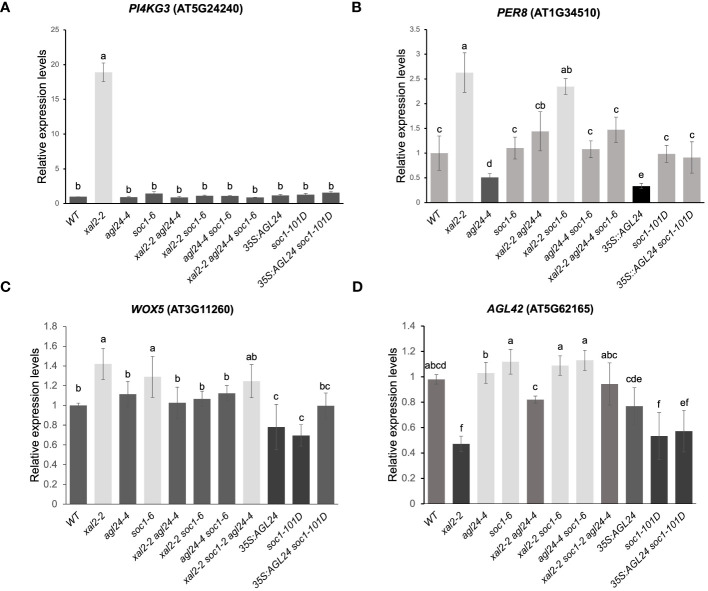
*XAL2, AGL24* and *SOC1* regulate the expression of different genes involved in salt/osmotic stress responses, cell wall expansion and QC identity. *PI4KG3* **(A)**, *PER8* **(B)**, *WOX5* **(C)** and *AGL42* **(D)**. Expression levels in roots from 7 dps plants of WT, *xal2-2*, *agl24-4*, *soc1-6*, the double mutants *xal2-2 agl24-4*, *xal2-2 soc1-6*, *agl24-4 soc1-6*, the triple mutants *xal2-2 agl24-4 soc1-6, 35S::AGL24, soc1-101D* and the double overexpression line *35S::AGL24 soc1-101D*. Data are shown as the mean ± SE of three independent biological replicates with two technical replicates. Letters indicate significant differences among the different lines (Student’s t-test or Wilcoxon Mann-Whitney test). *RNAH*, *PDF2* and *UPL7* were used as reference genes.

*PER8* was shown to be upregulated in the *xal2-2* and in *xal2-2 soc1-6.* The *PER8* expression levels were reverted to WT levels in the double and triple mutants *xal2-2 agl24-4*, *agl24-4 soc1-6* and *xal2-2 agl24-4 soc1-6* (**Figure 6B**). Also, the expression levels of this gene were lower in *35S::AGL24* and *agl24-4*, suggesting that AGL24 regulates *PER8* in a non-linear manner as both the loss or gain of function of *AGL24* downregulates the expression of this gene. Furthermore, *WOX5* expression levels are significantly lower in the overexpression lines *35S::AGL24* and *soc1-101D* (**Figure 6C**) and *AGL42* levels are lower in *soc1-101D* and *35S::AGL24 soc1-101D* (**Figure 6D**).

Notably, we found a drastic upregulation of the expression of *PI4KG3* in *xal2-2* that was completely reverted to WT levels in the double mutants *xal2-2 agl24-4* and *xal2-2 soc1-6* (**Figure 6A**). Moreover, the higher expression levels of *WOX5* as well as the lower *AGL42* expression levels in *xal2-2* were also reverted to WT levels in the double mutants *xal2-2 agl24-4* and *xal2-2 soc1-6*. These results suggest that the absence of either *AGL24* or *SOC1* is sufficient to revert the altered expression of *PI4KG3*, *WOX5* and *AGL42* to WT levels in the *xal2-2* background (**Figures 6A-D**).

## 3 Discussion

### 3.1 The flowering inducer MADS-box genes *XAL2*, *SOC1* and *AGL24* are expressed in root tissues

Our results reveal that *XAL2*, *SOC1* and *AGL24*, three important components in the GRN for floral transition, are expressed in root tissues and regulate different processes of root growth and development. Interestingly, we found that the SOC1 protein is localized in the SCN (**Figure 1C**), suggesting it has important roles in coordinating cell division and differentiation processes. Some other MADS-box genes such as *AGL16*, *AGL17*, *AGL21* and *AGL42* are expressed in this zone as well (Burgeff et al., 2002; Nawy et al., 2005; Yu et al., 2014; Zhao et al., 2021), so their proteins could potentially interact with SOC1 *in planta* as proven by yeast-two-hybrid assays (De Folter et al., 2005). Possible new protein interactions *in vivo* between SOC1 and other MADS-domain proteins localized in root tissues remain to be investigated. The pattern of *SOC1* expression and protein accumulation is highly similar in the root tip (**Figure 1C**, **Supplementary Figure 2B**). However, significant changes were observed when comparing the GUS pattern in the proximal region of the primary root, close to the hypocotyl, where an intense GUS signal is observed only in the transcriptional construct, but not in the translational construct (**Supplementary Figures 2C, D**). Further analyses are required to determine whether this contrasting pattern of *SOC1* expression versus protein accumulation is attributable to intronic regulatory elements or protein-level regulation of SOC1.

We observed that the pattern of AGL24 protein localization found in this study (**Figure 1B**; **Supplementary Figures 3C, D**) is different from the *AGL24* expression pattern when only the promoter region is used in the genetic construct (*pAGL24::GUS)*, in which an abundant GUS expression is localized in the vascular tissues of roots and leaves (**Supplementary Figure 3B**), which is the same as the previously reported by Huang et al. (2024). These huge differences between the pattern of gene expression and protein localization could be explained by the fact that the *AGL24* transcript is able to move long distances to act non-cell autonomously, and also, its protein is constantly being degraded via proteasome, so it is only accumulated in tissues where it exerts its function, as previously demonstrated by Huang et al. (2024). Interestingly, it has been reported that no AGL24-GFP signal could be detected in leaves or vascular tissues in transgenic plants carrying the *35S::AGL24-GFP* or *pSUC2::AGL24-GFP* constructs, but the AGL24-GFP signal was detected in the flower meristems, even when the *SUC2* promoter does not confer expression in this tissue (Huang et al., 2024). Similarly, in 7 dps plants, we did not detect the AGL24-GUS protein in leaf or root vascular tissues of plants carrying the *pAGL24::AGL24-GUS* translational construct (**Supplementary Figure 3C**), but we found a AGL24-GUS signal in the SAM and differentiated columella cells in several independent transgenic lines in a WT background as well as in the *agl24/pAGL24::AGL24-RFP* complementation line (**Supplementary Figure 3D**) (Gregis et al., 2009). This suggests that *AGL24* plays a role in the gene regulation in both the SAM and columella cells, as the transcripts travel long distances and the translated AGL24 protein is not degraded in these tissues.

Additionally, we found that the pattern of XAL2 protein localization differs from the previously reported gene expression pattern observed using either a 1-Kbp promoter-GUS fusion and the previous localization of *XAL2* transcript using *in situ* hybridization (**Supplementary Figure 1B, C**; Garay-Arroyo et al., 2013). These differences could be attributed to the differences in the promoter length: 1127 bp (Garay-Arroyo et al., 2013) vs 2792 bp (this study) (**Supplementary Figure 1A**), by the presence of other regulation regions not included in our constructs such as the 3’ UTR and distant enhancers or by the presence of intronic regulatory sequences in our translational construct. Intronic sequences are known to play important roles in the expression of several MADS-box genes, as previously reported for *AG*, *STK*, *SEP3*, *AGL6*, and *AGL13* (Sieburth and Meyerowitz, 1997; Deyholos and Sieburth, 2000; Kooiker et al., 2005; De Folter et al., 2007; Schauer et al., 2009; Singer et al., 2010). The expression patterns of these genes differ when only the ATG upstream regions are considered compared to when the whole genomic regions, including the introns, are considered (Sieburth and Meyerowitz, 1997; Deyholos and Sieburth, 2000; Kooiker et al., 2005; De Folter et al., 2007; Schauer et al., 2009; Singer et al., 2010). Further studies are necessary to unravel the function of intronic sequences in the regulation of these MADS-box genes in roots.

Furthermore, the *XAL2* transcripts have been reported to be cell-to-cell mobile (Thieme et al., 2015) and mobile mRNAs are transcribed in the source cells, but exert their function in specific recipient cells. However, in source cells, mobile mRNAs can be translated into proteins, but the translation of mobile mRNA-encoded TFs in the source cells may ectopically activate their downstream target genes. To maintain functional specificity of mobile mRNAs, proper translational or post-translational control of mobile mRNA in the source cells is necessary (Huang et al., 2024). Also, it could be possible for the XAL2 protein to undergo selective degradation processes in the primary root, similarly to what has been reported for AGL24 in leaves (Huang et al., 2024). Whether this mechanism is shared by other MADS-box genes encoding mobile mRNAs remains to be elucidated.

We detected the XAL2 protein in lateral roots of both *pXAL2::XAL2-GUS* in a WT background as well as in the *xal2-2 pXAL2::XAL2-GFP* complementation line (**Figures 1D**; **Supplementary Figures 1C**, **4F, G**). In addition, in the complementation lines, we found *XAL2* expression levels (**Supplementary Figure 4B**) and a root length with no significant differences when compared with the WT (**Supplementary Figures 4C, D**). This shows that our constructs have the sufficient elements to express *XAL2* at similar levels to those in WT plants and this expression is sufficient to recover the short-root phenotype of the *xal2-2* mutant. Additionally, the overexpression of *XAL2* caused a long-root phenotype (**Supplementary Figures 4C, D**), which confirms again the role of *XAL2* as a promoter of root growth.

Unexpectedly, we could not detect the presence of the XAL2 protein in the primary root meristems (**Supplementary Figures 1C**, **4E**), even though the loss-of-function mutants of this gene display conspicuous primary root phenotypes (Garay-Arroyo et al., 2013). Some other genes involved in the auxin homeostasis regulation in roots such as *GH3.6*, *GH3.6* and *GH3.9* have been reported to exhibit undetectable GFP signals in primary roots when analyzing protein-GFP translational fusions, despite a YFP signal being visible in primary roots of plants carrying promoter-YFP transcriptional fusions (Pierdonati et al., 2019).

Finally, *XAL2* is reported to have at least four alternative splicing variants, with two of them retaining intronic sequences (The Arabidopsis Information Resource (TAIR), 2024). Consequently, these variants are predicted to form premature stop codons, resulting in truncated proteins. Since we fused the GUS protein to the C-terminal region of XAL2, we will not be able to visualize other splice variants that encode truncated proteins using our constructs. The function of the different splice variants of *XAL2* in plant development has yet to be investigated.

### 3.2 SOC1 and AGL24 as negative regulators of root growth and cell size

*SOC1* and *AGL24* single mutants did not exhibit evident root phenotypes. However, when examining double and triple mutant combinations, along with overexpression lines, we discovered root phenotypic changes that affected primary root growth and cell differentiation processes. Consequently, our findings suggest that *SOC1* and *AGL24* not only act redundantly as repressors of primary root growth, but their loss of function restores the primary root growth, meristem cell number, and the length of fully elongated cells in the *xal2-2* mutant background. Furthermore, these results demonstrate that XAL2 and either SOC1 or AGL24 are involved in primary root growth inhibition through different pathways. Given these findings, it could be interesting to compare the transcriptome among the different single mutants such as *agl24-4* or *soc1-6* with the double mutants *xal2-2 agl24-4* or *xal2-2 soc1-6* to find more genes that participate either as suppressors or promoters of Arabidopsis primary root growth.

### 3.3 SOC1 functions as a negative regulator of columella differentiation

Lugol staining analyses in the loss- and gain-of-function mutants, suggest that *SOC1* might act as a negative regulator of columella cell differentiation as the *soc1-6* mutant displays a significantly different frequency of the DCC layers and the *soc1-101D* overexpression line shows a drastic change in the proportion of both CSC and DCC layers. Previous studies have reported that SOC1 requires interaction with AGL24 to be translocated to the nucleus (Lee et al., 2008). Given their co-localization pattern in the very apical layer of columella cells (**Figure 1E**), it is likely that they could potentially interact in this tissue. Further experiments are needed to prove if SOC1-AGL24 protein-protein interactions occur *in vivo*, specifically, in root tissues and it will be interesting to elucidate the composition of the protein complexes that participate in these phenotypes.

### 3.4 The mutual regulation of SOC1 and AGL24 expression differs between aerial tissues and the roots

It has been demonstrated that SOC1 and AGL24 proteins interact and form a heterodimer that activates many of their target genes; moreover, they directly and positively regulate each other’s expression in seedlings (Liu et al., 2008). Interestingly, our data indicate a negative regulation of *AGL24* expression by SOC1. Since MADS-domain proteins exert their function as tetramers to activate or repress the expression of their target genes, the differential regulation observed in roots could be attributed to tissue-specific variations in the composition of the MADS-domain protein tetramers with distinct DNA binding specificities, or the presence of a negative regulator of these genes in roots. It could be interesting to explore these two possibilities in the Arabidopsis roots. Dynamic changes of protein complex formation on specific tissues have been described for the MADS-domain protein FRUITFULL (FUL) (van Mourik et al., 2023), suggesting that other MADS-domain TFs might exhibit contrasting gene regulation of their target genes in a tissue-specific manner.

### 3.5 New roles of XAL2 in root development and response to stress conditions

Through our transcriptome analyses, we found that different genes involved in osmotic and salt stress responses, as well as cell wall organization and biogenesis are regulated by XAL2. These findings collectively point towards significant roles that XAL2 potentially fulfills in abiotic stress responses and cell elongation processes.

*DEFL207* (AT5G33355), a gene belonging to the DEFENSIN-LIKE (DEFL) gene family, was downregulated in *xal2-2* (**Supplementary Figure 9B**). *DEFL207*, along with other closely related DEFL genes such as *DEFL202*, *DEFL203*, *DEFL206* and *DEFL208*, are reported to be Zn-deficiency-responsive genes (Kimura et al., 2023). The specific function of *DEFL207* is yet unknown. However, it is worth noting that its relatives *DEFL202* and *DEFL203* are involved in the inhibition of root growth under Zn-deficient conditions through a reduction in root meristem length and cell number (Kimura et al., 2023).

Another group of genes enriched in our RNA-seq data included enzymes such as xyloglucan endotransglucosylases/hydrolases (XTHs) and peroxidases, two classes of enzymes that play important roles in plant cell wall organization and biogenesis. XTHs are enzymes that contribute to the modification of the xyloglucan component in the plant cell wall. These enzymes facilitate cell wall relaxation during growth by loosening the connections between cellulose microfibrils and xyloglucan chains. This allows the cell to expand without rupturing the cell wall. Importantly, XTHs are responsive to various stresses and might play roles in cell wall reinforcement during stress conditions (Ishida and Yokoyama, 2022). Specifically, we found upregulation of *XTH13* and *XTH26* in *xal2-2* (**Supplementary Figure 9A**).

Dirigent proteins (DIRs) and peroxidases have frequently been implicated in modulation of lignification levels upon exposure to abiotic stress. The expression of several of the DIR-like genes was reported to be responsive to water, abscisic acid (ABA), and cold stress (Arasan et al., 2013; Paniagua et al., 2017). Notably, in the context of water stress, the expression of most DIR genes appears to be correlated with increased lignification (Arasan et al., 2013; Paniagua et al., 2017). In our study, we found that *DIR20* was downregulated in *xal2-2* (**Figure 5C**; **Supplementary Figure 9B**), suggesting that XAL2 could be involved in regulating lignin biosynthesis processes as well as abiotic stress responses. Peroxidases, on the other hand, contribute to cell wall organization and biogenesis through cross-linking of lignin, cell wall reinforcement and ROS regulation (Francoz et al., 2015; Raggi et al., 2015; Jemmat et al., 2020; Jeong et al., 2022).

In our RNA-seq dataset, we observed an upregulation of *PER8* and *PER52* in *xal2-2* (**Figure 6B**; **Supplementary Figure 9A**). Furthermore, the expression of *PER8* was found to be regulated by *AGL24* in a non-linear manner, as both the loss of function and overexpression of this gene lead to a downregulation of this *PER8*. Notably, the elongated root phenotypes observed in the *35S::AGL24* overexpression line (**Figure 2A**) and *agl24-3* mutant line (**Supplementary Figure 6B**) might be partially explained by the diminished expression of *PER8*. Previous research has shown that *PER8* is a repressor of primary root growth (Jeong et al., 2022). Interestingly, the expression of *PER8* in roots is upregulated in *xal2-2* and *xal2-2 soc1-6*, while it is downregulated in *agl24-4*. However, in the double mutant *xal2-2 agl24-4*, the expression of *PER8* returns to that of the WT (**Figure 6B**). In this case, *AGL24* acts as an antagonist to *XAL2* in the regulation of *PER8*, whereas *SOC1* does not. This observation strongly suggests that *AGL24* is partially redundant with *SOC1*, as it regulates distinct pathways.

Additionally, we found that these three MADS-box genes are important for maintaining root stem cell homeostasis because their loss of function affects the expression of QC-specific genes such as *WOX5* (Sarkar et al., 2007), *AGL42* (Nawy et al., 2005), and *NTT/WIP2* (Crawford et al., 2015) (**Figures 6C, D**; **Supplementary Figure 9C**). Interestingly, we observed a correlation between low expression levels of *AGL42*, and short root primary phenotypes found in *xal2-2* and in the double mutants *xal2-2 agl24-4* and *35S::AGL24 soc1-101D*. To the best of our knowledge, there is no information on the role of *AGL42* in primary root growth and this association suggests that this gene could participate in primary root growth. It was reported that SOC1 directly binds to the *AGL42* intron sequences to regulate its transcription in aerial tissues (Dorca-Fornell et al., 2011), so this gene could also be a direct target of SOC1 in roots. A comprehensive analysis using *AGL42* mutants and overexpression lines is needed to uncover its role in root development and cell differentiation.

We found that *PI4KG3* expression levels increase dramatically in the *xal2-2* mutant in both the RNA-seq and RT-qPCR data (**Figure 6A**; **Supplementary Figure 9B**). This gene is upregulated in response to high salinity conditions, drought, cold, heat, or ABA treatments (Akhter et al., 2016). *PI4KG3* overexpression lines display enhanced tolerance to high salinity or ABA in comparison to WT plants (Akhter et al., 2016), suggesting that the absence of *XAL2* is sufficient to trigger a salt and osmotic stress response, leading to high *PI4KG3* expression levels.

In summary, XAL2 is an important component for the regulation of several gene groups, including osmotic stress- and salt-responsive genes, XTHs, peroxidases, and QC-specific TFs. Together, these genes contribute to the dynamic and adaptable nature of the primary root growth under different conditions.

## 4 Concluding remarks

Our study confirms that these three MADS-box genes (*SOC1*, *AGL24* and *XAL2*) are components of a GRN involved in cell proliferation and cell differentiation in the primary root. We demonstrated that *SOC1* and *AGL24*, which are critical components in flowering transition, redundantly act as *XAL2* antagonists, as their absence recovers the primary root growth, meristem cell number, cell production rate, and the length of fully elongated cells in the short-root mutant *xal2-2*. Moreover, we also reported that the expression of some genes (*PI4KG3, WOX5* and *AGL42*) in *xal2-2* returned to WT levels in the double mutants *xal2-2 agl24-4* and *xal2-2 soc1-6*.

Interestingly some MADS-box genes have been documented to play a role in stress responses during primary root growth, such as *AGL16* (Zhao et al., 2020, 2021). Our RNA-seq analysis also showed that *XAL2* is an important regulator for several genes involved in stress responses. It is worth highlighting that the primary root length in *AGL16* loss- and gain-of-function plants is not different from that of WT plants under control conditions. However, this gene acts as a negative regulator of root growth under different types of stress (Zhao et al., 2021). Thus, it is very plausible that many of the MADS-box genes that are expressed in roots with no apparent phenotypes in primary roots growing in control conditions like *SOC1* and *AGL24*, could participate in different stress responses as was demonstrated for *AGL16* (Zhao et al., 2021). A comprehensive expression analysis of all of the Arabidopsis MADS-box genes expressed in roots under both control and different stress conditions, as well as their phenotypes, could uncover many novel biological functions of these genes in root development.

Future work should incorporate the examination of the various genes that are upregulated and downregulated in all the single and double mutants utilized in this study. This exploration aims to identify genes that may play a role in primary root development. Subsequently, it will be interesting to explore the composition of the different protein complexes formed in these mutant backgrounds. This step will contribute to uncovering novel biological functions of these genes in root development.

## 5 Materials and methods

### 5.1 Plant growth conditions

All experiments and lines in this work are in a Col-0 background and homozygous lines of *agl24-4* (GK674F05.3/N385337, Pérez-Ruiz et al., 2015), *soc1-2* (Balanzà et al., 2014), *soc1-6* (Salk_138131; Pérez-Ruiz et al., 2015), *xal2-2* (Garay-Arroyo et al., 2013), *35S::AGL24* (Yu et al., 2002; Pérez-Ruiz et al., 2015), *soc1-101D/AGL20-101D* (Lee et al., 2000; Pérez-Ruiz et al., 2015), *agl24/pAGL24::AGL24-RFP* (Gregis et al., 2009) and the double mutants *xal2-2 agl24-4* and *xal2-2 soc1-6* (Pérez-Ruiz et al., 2015) were used. Seeds of *agl24-3* (Salk_095007C) were provided by the Arabidopsis Biological Resource Center or the Nottingham Arabidopsis Stock Centre. We generated the following double and triple mutants: *xal2-2 agl24-3*, *xal2-2 soc1-2*, *agl24-4 soc1-6*, *35S::AGL24 soc1-101D* and *xal2-2 agl24-4 soc1-6*.

Plants were grown in 0.2X Murashige-Skoog (MS) salt plant media (MP Biomedicals, cat #2633024), 0.5 g/L MES (Sigma-Aldrich), 1% sucrose (Sigma-Aldrich), 1% Bacto Agar (Becton, Dickinson and Company) and adjusted to pH 5.6 with KOH. Seeds were disinfected in rotation with absolute ethanol for 5 minutes followed by a 13-minute treatment with a solution containing 1% SDS and 5% chlorine. Subsequently, they were rinsed four times with sterile water. The seeds were stratified at 4°C for 2-5 days under dark conditions and sown on square Petri dishes containing MS medium. The plates used for primary root growth and RT-qPCR assays were placed vertically in growth chambers kept at 21-23.5°C with a long day photoperiod (16-h light/8-h dark) and a light intensity of 110 μmoles m^-2^s^-1^.

The different lines were grown for 5 days in vertical petri dishes and then transferred to fresh media to prevent over-evaporation of the cultures during a 12-day timelapse. The primary root growth was measured for 7 days, starting from the day of transplantation (dpt; day 5). The tip of the root was marked after 5 dps every day at the same hour, and roots were scanned and measured using the ImageJ software. We grew six seedlings per plate and conducted three independent replicates, each consisting of n= 30 seedlings per mutant line. The data for all of the root length measurements is available in **Supplementary Table 8**.

For statistical analyses of root length, normality was conducted using Kolmogorov-Smirnov tests. For multiple comparisons, a Kruskal-Wallis test followed by Dunn’s *post-hoc* test was conducted.

### 5.2 Plasmid constructs and selection of transgenic plants

Genomic DNA from Arabidopsis ecotype Columbia (Col-0) was used as the template for amplification. All constructs were generated using the Gateway system (Invitrogen) for in-frame GUS fusion in the C-terminal region of SOC1, AGL24 and XAL2, as well as promoter-GUS/GFP fusions. For the *pSOC1::SOC1-GUS* translational fusion, a 4906 bp *SOC1* genomic region was amplified from region -2516 to region +2392, omitting the stop codon using primers pSOC1ND FW and SOC1MA-R (primers sequences are found in **Supplementary Table 6**). The PCR product was cloned into the pCR8/GW/TOPO entry vector (Invitrogen; K250020). Subsequently, the *pCR8/GW/TOPO/pSOC1::SOC1* plasmid was recombined into the *pGWB3* destination vector (Nakagawa et al., 2007) via LR reaction using LR Clonase II Plus enzyme (Invitrogen).

For the *pAGL24::AGL24-GUS* translational fusion, a previously reported plasmid construct *pB7RWG2/pAGL24::AGL24:RFP* was used (Gregis et al., 2009). The plasmid was recombined back into the entry vector *pDONR-207* via a BP reaction using BP Clonase (Invitrogen) and the resulting plasmid *pDONR-207/pAGL24::AGL24* was recombined into the destination vector *pGWB3* (Nakagawa et al., 2007).

To generate the *pXAL2::XAL2-GUS* translational construct, a genomic *XAL2* region was PCR amplified from position -2 to position +3594, omitting the stop codon using primers AGL14F19A and OAGR2 (see **Supplementary Table 6**). We used a forward primer that adds an *AscI* restriction site in position -2 and a reverse primer without any additional site. The 3604 bp genomic fragment amplified by PCR was cloned into the pCR8/GW/TOPO entry vector, thus generating the *pCR8/GW/TOPO/XAL2g* plasmid. As it was not possible to amplify the *XAL2* promoter region using PCR, a 2792 bp region upstream the *XAL2* ATG was synthesized and flanked with *AscI* sites (Gene Universal, DE, USA). The synthesized promoter region was ligated into the previously described *pCR8/GW/TOPO/XAL2g* plasmid in the AscI site (located at position -2 upstream the *XAL2* start codon) to generate the *pCR8/GW/TOPO/pXAL2::XAL2* plasmid, which was subsequently recombined into the pGWB3 or pGWB4 vectors (Nakagawa et al., 2007) for fusion with GUS or GFP, respectively, via an LR reaction.

For *pXAL2*, *pAGL24* and *pSOC1* transcriptional constructs, the *XAL2*, *AGL24* and *SOC1* 5’ upstream regions (1127 bp, 2089 bp and 2515 bp, respectively) were amplified using primers AGL14F5P and PX2-HDNA-RV for *XAL2* promoter region; AGL24MA-F and AGL24MA-R for *AGL24* promoter region; and pSOC1ND FW and pSOC1ND-R for *SOC1* promoter region (see **Supplementary Table 6** for primer sequences). The PCR-amplified regions were subsequently cloned in the pCR8/GW/TOPO entry vector (Invitrogen; K250020) and then recombined in the pGWB3 or pGWB4 destination vectors for GUS or GFP fusions, respectively.

All constructs were introduced into *Agrobacterium tumefaciens* strain C58 by electroporation, and Arabidopsis WT (Col-0) plants were transformed using the floral dip method (Zhang et al., 2006). The transgenic lines were selected with kanamycin (50mg/L) on MS plates and analyses were carried out on T3 homozygous lines. At least four independent transgenic lines were analyzed for GUS/GFP localization in root tissues and the representative patterns were presented.

### 5.3 GUS histochemical assay

GUS histochemical assay was performed as described in Perilli and Sabatini (2010). Briefly, 7 dps *pSOC1::SOC1-GUS*, *pAGL24::AGL24-GUS, pXAL2::GUS* and *pXAL2::XAL2-GUS* plants were incubated on X-Gluc solution (100 mM Na_2_HPO4, 100 mM NaH_2_PO4, 0.5 mM K_3_Fe(CN)_6_, 0.5 mM K_4_Fe(CN)_6_, 0.1% Triton X-100 and 0.5 mg/ml X-gluc.) for 16 h at 37°C in the dark. For *pSOC1::GUS* roots, plants were incubated only for 40-60 minutes. After the incubation, plants were washed three times with distilled water. The chlorophyll in stained plants was eliminated through three washes with 96% ethanol. Subsequently, the plants were hydrated in a solution containing 30% glycerol and 2% DMSO for 24-48 h. The plants were mounted in a solution containing 70% chloral hydrate and 20% glycerol for visualization under microscopy.

### 5.4 Pseudo Schiff staining

To measure root cell size in plants grown under control conditions, seedlings were stained with a modified Truernit protocol (Truernit and Haseloff, 2008) as described in Cajero-Sánchez et al. (2019): 6 dps seedlings were fixed in 50% ethanol and 10% acetic acid at room temperature for 5 hours or overnight. After fixation, roots were washed three times with distilled water and then incubated for 50 minutes in 1% periodic acid at 37°C. Afterwards, seedlings were again washed for three times with distilled water and then placed for 50 minutes in 100 mM sodium metabisulfite, 0.15 N hydrochloric acid, and 15 μg/mL propidium iodide at room temperature for 2 h. Treated seedlings were washed three times with distilled water and later were submerged in a solution with 2% DMSO and 30% glycerol for 72 hours. For microscopy observation, plants were mounted in a sodium iodide solution (20 mL of 65% glycerol, 2% DMSO, with 0.04 g of sodium thiosulfate and 17 g of sodium iodide).

### 5.5 Microscopy visualization

Seedlings mounted with pseudo-Schiff staining were observed by microscopy (40x, Olympus BX60 microscope with Nomarski optics; Tokyo, Japan) to measure the length of the cortex cells. *In vivo* observation for meristematic zone measurements was done by staining with 10 ng/mL propidium iodide and confocal images were acquired with a Nikon A1R+ with a dry X20 objective laser scanning confocal head coupled to an Eclipse Ti-E inverted microscope (Nikon Corporation, Tokio, Japan) and Nis Elements C v.5.00 software was used. Single plane images were captured using GaAsP galvanometric scanners and excitation wavelengths 561 nm (red light emission).

### 5.6 Analysis of cellular parameters of root growth

Cell length measurements were done using ImageJ software. A minimum of nine roots per treatment were measured in each case using cortical cell length from the QC until 10 cells after the first epidermal hair root. The RAM cell number and length domains were obtained with the web tool of multiple structural change algorithm for cell root analysis: www.ibiologia.com.mx/MSC_analysis (Pacheco-Escobedo et al., 2016), and the length of the fully elongated cells was obtained with the mean value from 9 or more cells after the cortical cell nearest to the epidermal cell with the first hair root. The cell production rate (cells/h) was calculated as V/(le), where V (μm/h) is the root growth rate during the last day of growth (from day 5 to 6), and le (μm) is the average length of, at least, 10 fully elongated cells. Cellular measurements were assessed for normality using the Shapiro-Wilks test (1965) (Shapiro and Wilk, 1965). If the data were found to be normally distributed, they were compared using one or two-way analysis of variance (ANOVA) followed by a Tukey’s *post-hoc* test. Additionally, we examined the correlation between primary root length and meristem cell number, fully elongated cell size, and cell production rate using the Pearson correlation test.

### 5.7 Root stem cell niche morphological analysis

We analyzed the proportion of columella stem cells (CSC) and differentiated columella cells (DCC) in 90 plants from the different mutant lines. The analysis involved treating 5 dps or 6 dps Arabidopsis roots with Lugol for two minutes and mounting them in a clearing solution (80% chloral hydrate and 20% glycerol). The mounted roots were then visualized using an Olympus BX60 microscope (Olympus, Tokyo, Japan) equipped with Nomarski optics. Subsequently, the obtained data were subjected to statistical analysis using a Fisher test followed by Bonferroni correction for multiple testing using RStudio to assess the significance differences observed among the mutant lines.

### 5.8 RNA extraction and RT-qPCR procedures

To determine the relative gene expression, 7 dps plants of the different lines were grown under control conditions. Root total RNA was extracted from three independent biological replicates, with each replicate consisting of approximately 100 plants. The plant tissues were rapidly frozen in liquid nitrogen and stored at -70°C. RNA extraction was performed using the Quick-RNA Miniprep kit (Zymo Research). Subsequently, 2 μg of total RNA was reverse transcribed with SuperScript III Reverse Transcriptase (Invitrogen) following the manufacturer’s instructions. The resulting cDNA was diluted 20-fold, and 400 μL of cDNA were obtained from 2 μg of RNA. The qPCR reactions were carried out using 5 μL of Maxima SYBR Green qPCR Master Mix (Thermo Scientific), forward and reverse primers at a final concentration of 0.2 μM, and 2 μL of template cDNA, resulting in a total reaction volume of 10 μL. Non-template controls (NTCs) and samples were analyzed in triplicate. All qPCR reactions were conducted in a StepOne real-time PCR system (Applied Biosystems). The program used was: 2 min at 50°C, 10 min at 95°C; 40 cycles of 95°C for 15 sec and 60°C for 30 sec; followed by a melting curve (60°C-95°C).

The PCR amplification efficiency of each gene and individual CT’s were calculated using LinRegPCR software (Ruijter et al., 2009). The relative expression was calculated with the E^-ΔΔCT^ method (Rao et al., 2013), using three biological replicates, with three technical replicates each, and we used *RNAH*, *PDF2* and *UPL7* as housekeeping control genes (Czechowski et al., 2005; Hong et al., 2010), and WT as the control line. The statistical analysis was performed using the ΔCT values in RStudio using Student-T test for parametric data and Wilcoxon–Mann–Whitney assay for non-parametric data in pairwise comparisons. Primer sequences are included in **Supplementary Table 5**. For *XAL2, SOC1* and *AGL24* expression analyses in root and aerial tissues, the expression of these genes was compared with that of *RNAH*, *PDF2* and *UPL7* (ΔCT).

### 5.9 RNA-seq analysis

For RNA-seq analysis, we collected 7 dps Arabidopsis roots from WT (Col-0) and *xal2-2* (Garay-Arroyo et al., 2013). Then, three RNA biological replicates for each genetic background were isolated using the Quick-RNA Miniprep kit (Zymo Research). Total RNA integrity was evaluated using the Bioanalyzer (RIN ≥ 9.1). The RNAseq library was sequenced by NovoGene (Sacramento, CA, USA). The gene count determination was performed by NovoGene (Sacramento, CA, USA), including quality control per sequence which involved removing reads with adapters, poly-N and low quality reads.

The reference genome and the gene annotation file belong to TAIR10 with the latest update from 2010-09 and the accession number GCA_000001735.1 from The Arabidopsis Information Resource (TAIR). The index construction was built using Hisat v2.0.5 (Kim et al., 2019). The mapped reads were assembled with StringTie v1.3.3b (Pertea et al., 2015). And gene counts were obtained using featureCounts v1.6.5 (Liao et al., 2019).

The differential expression analysis was performed using DESeq2 v1.12.3 (Love et al., 2014). The normalization for the visualization of data distribution was performed using the estimateSizeFactors() and counts(dds, normalized= TRUE) functions from the same package. The results of differentially expressed genes (DEG’s) were generated with a significance alpha value of 0.05, using a threshold of (padj <0.05 and |log2 fold change| > 1.5). Gene ontology (GO) term enrichment analysis for each DEGs was conducted using agriGo (http://systemsbiology.cau.edu.cn/agriGOv2/index.php), ShinyGo (http://bioinformatics.sdstate.edu/go/) and DAVID bioinformatics (https://david.ncifcrf.gov/) tools.

## Data availability statement

The datasets presented in this study can be found in online repositories. The names of the repository/repositories and accession number(s) can be found below: GEO accession number: GSE247158.

## Author contributions

CC-S: Methodology, Supervision, Validation, Writing – original draft, Writing – review & editing, Formal Analysis, Investigation. MA: Formal Analysis, Investigation, Methodology, Project administration, Supervision, Validation, Writing – original draft, Writing – review & editing. NC-M: Formal Analysis, Investigation, Methodology, Writing – review & editing. DS-R: Methodology, Project administration, Writing – review & editing. CC-C: Data curation, Formal Analysis, Software, Visualization, Writing – review & editing. EZ-M: Formal Analysis, Methodology, Writing – review & editing. SP-O: Formal Analysis, Investigation, Methodology, Validation, Writing – review & editing. JA-G: Data curation, Formal Analysis, Software, Validation, Visualization, Writing – review & editing. BG-P: Funding acquisition, Resources, Supervision, Writing – review & editing. MS: Funding acquisition, Resources, Writing – review & editing. EÁ-B: Conceptualization, Funding acquisition, Resources, Writing – review & editing. AG-A: Conceptualization, Funding acquisition, Investigation, Methodology, Project administration, Resources, Supervision, Writing – original draft, Writing – review & editing.
