# Supplementary material for: The MADS-box genes SOC1 and AGL24 antagonize XAL2 functions in Arabidopsis thaliana root development
Source: Front Plant Sci. 2024 Mar 21;15:1331269. doi: 10.3389/fpls.2024.1331269 (PMC10994003; doi:10.3389/fpls.2024.1331269)
Supplement: Supplementary file 1 [file DataSheet_1.docx]

**SUPPLEMENTARY MATERIAL**


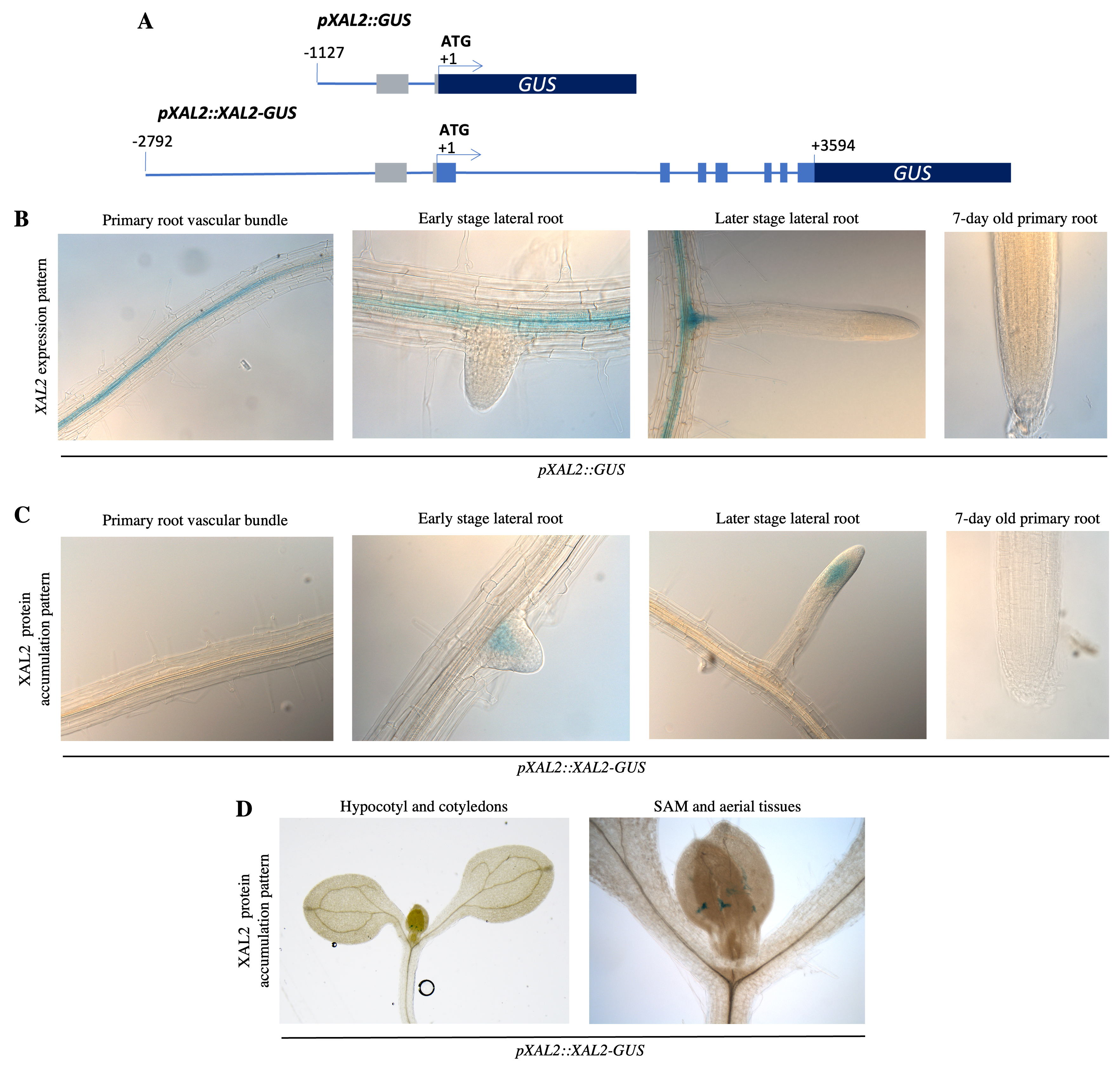


Supplementary Figure 1. **Patterns of *XAL2* expression and protein accumulation in 7 days-post-sowing (dps) plants.** (A) Schematic representation of the *pXAL2::GUS* transcriptional construct and *pXAL2::XAL2-GUS* translational construct. Gray boxes represent the 5’ UTR, blue boxes represent exons, and lines represent the intergenic regions and introns. (B) Expression pattern of *XAL2* in the roots of 7 dps plants carrying the promoter-GUS (*pXAL2::GUS*) transcriptional construct (16-hour incubation in X-gluc solution). XAL2 is expressed in the vascular bundle but not in the meristems of the primary or lateral roots. (C) In contrast, the XAL2 protein is exclusively localized in the meristems of lateral roots and is not detected in the primary root meristem or vascular bundle as observed in plants carrying the *pXAL2::XAL2-GUS* translational construct. (D) In aerial tissues, XAL2 is also accumulated in trichomes of early developing leaves. The *XAL2* expression patterns and protein accumulation patterns were observed in at least three independent transgenic lines. Representative pictures are shown (n=30).


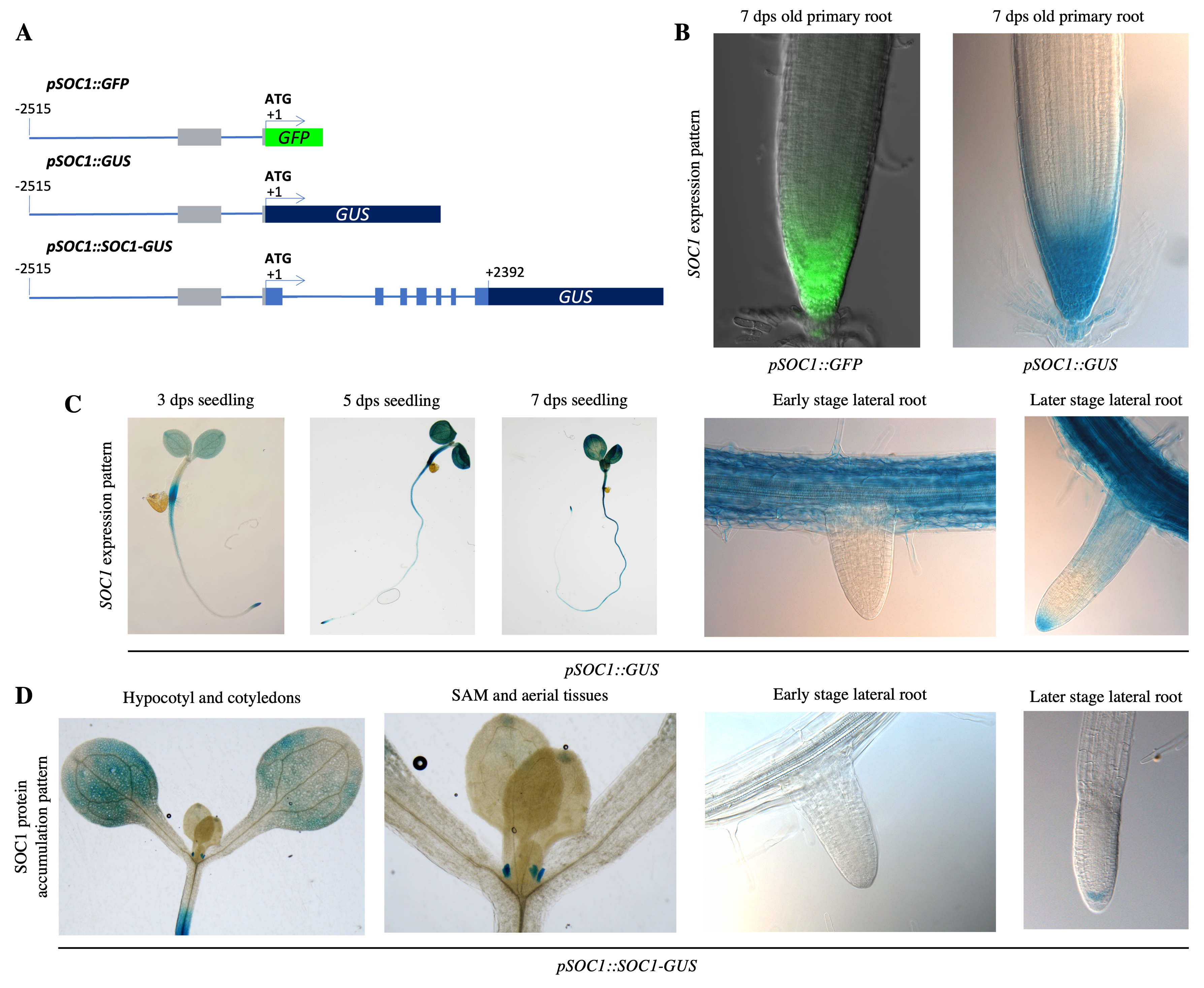


Supplementary Figure 2. **Patterns of *SOC1* expression and protein accumulation in different tissues in 7 dps plants.** (A) Schematic representation of the *pSOC1::GFP* and *pSOC1::GUS* transcriptional constructs, as well as the *pSOC1::SOC1-GUS* translational construct*.* Gray boxes represent the 5’ UTR, blue boxes represent exons and lines represent the intergenic regions and introns.  (B) Expression pattern of *SOC1* in 7 dps primary roots observed in plants carrying the *pSOC1::GFP* and *pSOC1::GUS* transcriptional constructs (40-minute-incubation in X-gluc solution). (C) *SOC1* expression pattern observed in 3, 5 and 7 dps seedlings as well as in early and later stages of lateral root development, as revealed by the *pSOC1::GUS* transcriptional fusion (16-h-incubation in X-gluc solution). (D) Localization of SOC1 protein in different tissues. In aerial tissues, SOC1 accumulates in the hypocotyl, cotyledons, and axillary shoot meristems, while in lateral roots, it is only detected in later stages of developing lateral roots. The *SOC1* expression patterns and protein accumulation patterns were observed in at least three independent transgenic lines. Representative pictures are shown (n=30).


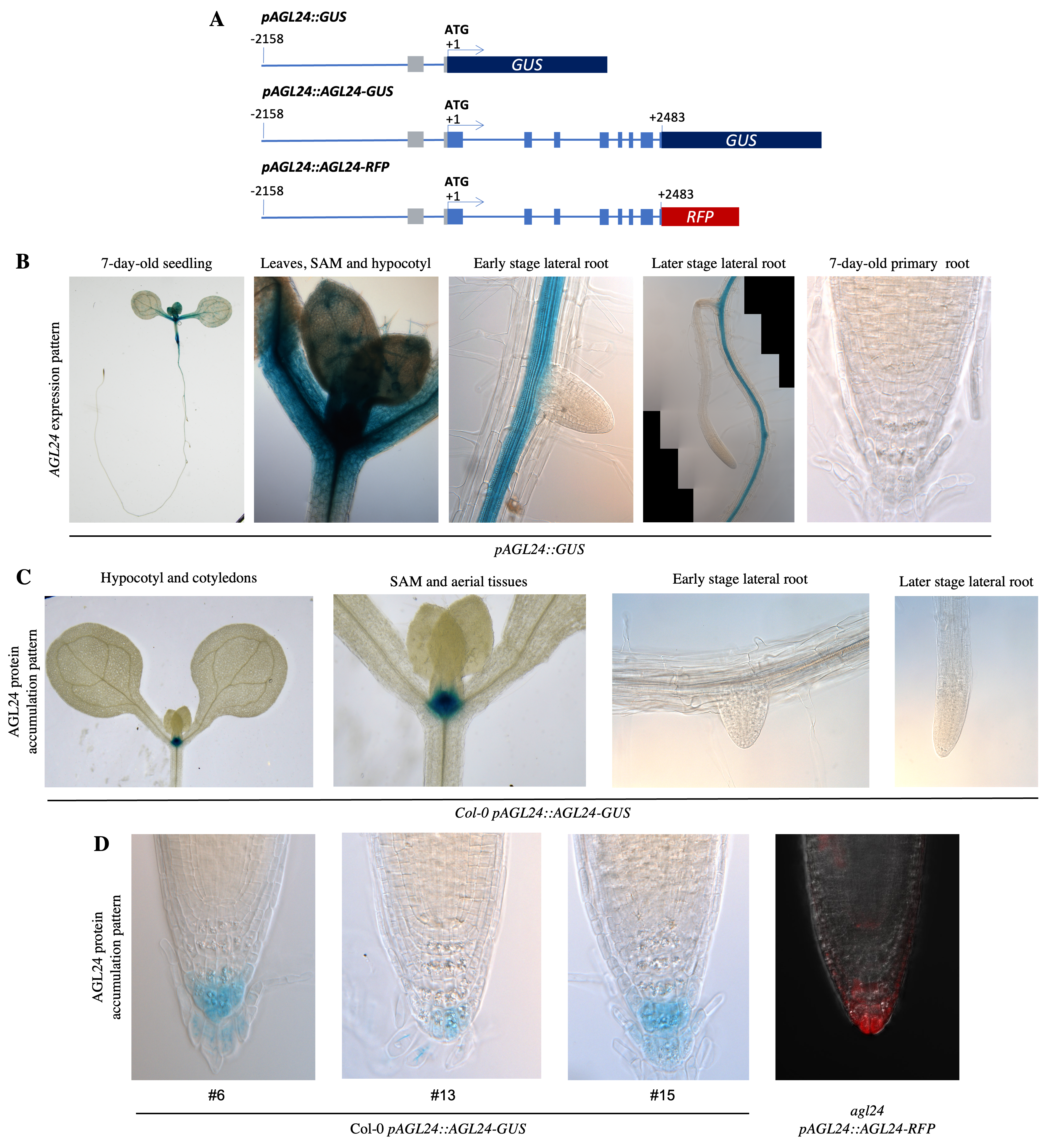


Supplementary Figure 3. **Contrasting patterns of *AGL24* expression vs protein accumulation in 7 dps roots.** (A) Schematic representation of the transcriptional construct *pAGL24::GUS* and the *pAGL24::AGL24-GUS* and *pAGL24::AGL24-RFP* (Gregis *et al*., 2009) translational constructs. Gray boxes represent the 5’ UTR, blue boxes represent exons and lines represent the intergenic regions and introns. (B) *AGL24* expression pattern observed in 7 dps seedlings, in the shoot apical meristem (SAM), whereas it is absent in early and later stages of lateral root development, and in the primary root, as revealed by the *pAGL24::GUS* transcriptional fusion (16-hour incubation in X-gluc solution). (C) In aerial tissues, the AGL24 protein is exclusively accumulated in the SAM, whereas it is not detected at any stage of developing lateral roots. (D) The AGL24 protein consistently accumulates in the differentiated columella cells. The patterns of AGL24-GUS protein accumulation are observed in at least three independent transgenic lines in a Col-0 background, and it is also observed in the *agl24 pAGL24::AGL24-RFP* complementation line. Representative pictures are shown (n=30).


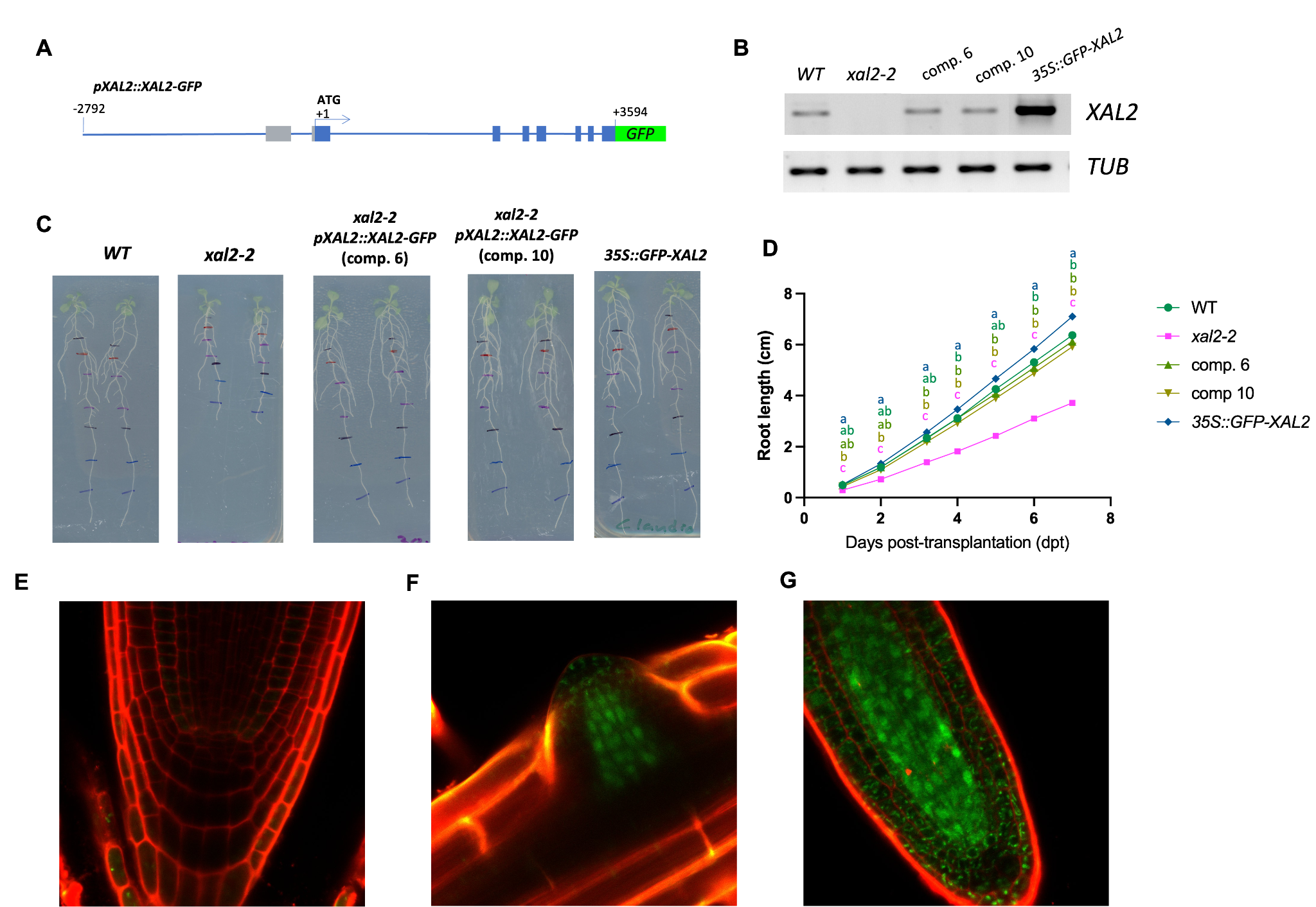


Supplementary Figure 4. **Complementation of the *xal2-2* mutant.** (A) Schematic representation of the construct *pXAL2::XAL2-GFP* which was used for complementing the *xal2-2* mutant. The full-length genomic region of *XAL2* was fused to the *GFP* gene at the 5’ of the corresponding last exon. Gray boxes represent the 5’ UTR, blue boxes represent exons and lines represent the intergenic regions and introns. (B) *XAL2* expression levels in WT, *xal2-2*, two independent complementation lines *xal2-2/pXAL2::XAL2-GFP* (lines 6 and 10) and WT/*35S::GFP-XAL2* as determined by semiquantitative RT-PCR. Root phenotypes (C) and primary root growth kinetics (D) of the mentioned lines. Representative pictures are shown (n=30). Colors and letters indicate significant differences as determined by Kruskal-Wallis test followed by Dunn’s *post-hoc* test. Pattern of the XAL2 protein localization observed in *xal2-2/pXAL2::XAL2-GFP* comp. 6 line. Primary root meristem (E), lateral root primordium (F) and lateral root meristem (G).


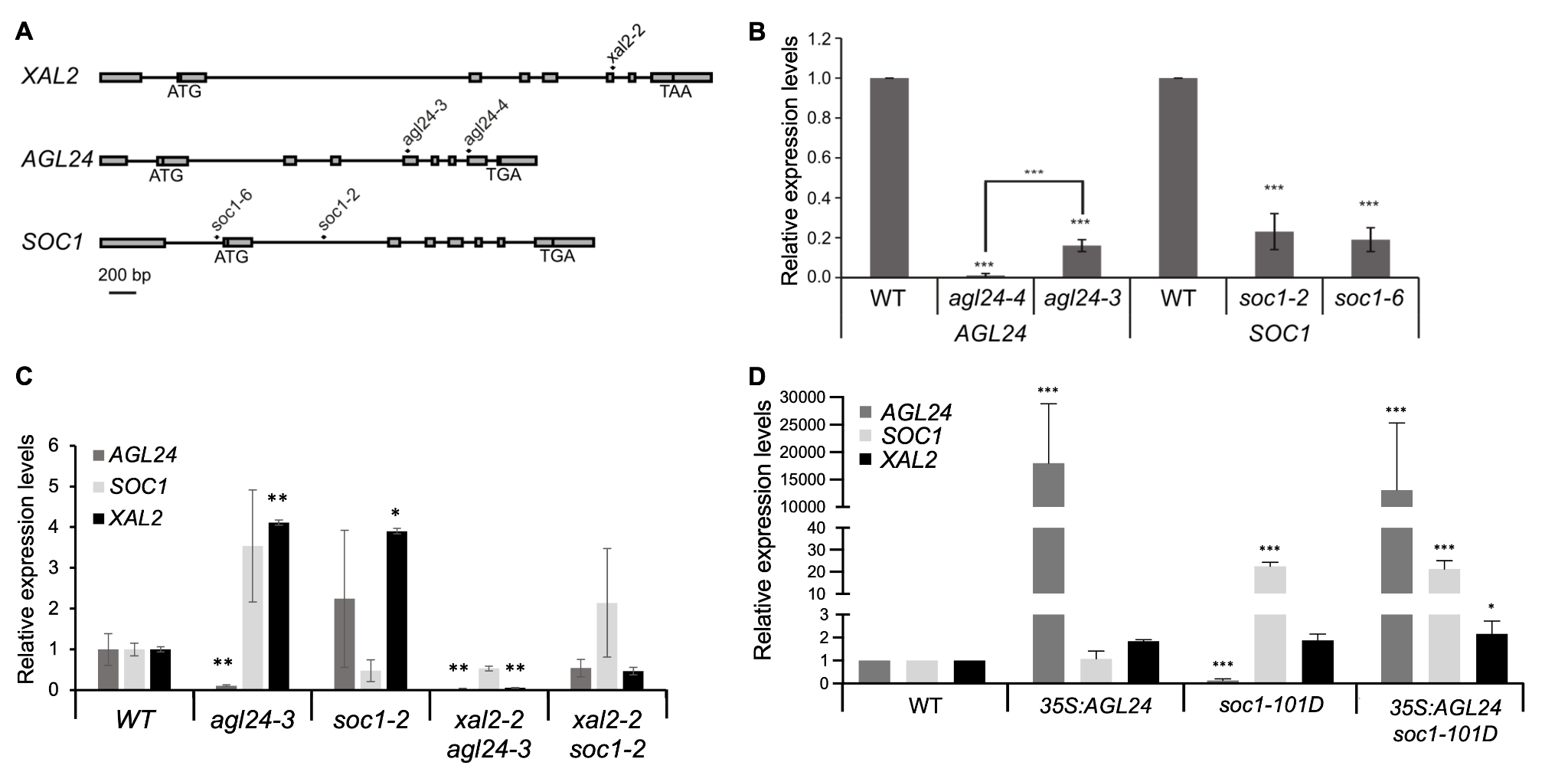


Supplementary Figure 5. **Expression of *AGL24* and *SOC1* and *XAL2* in different mutant lines and overexpression lines.** (A) *XAL2*, *AGL24* and *SOC1* gene structure schematic model with the sites of transposon (*xal2-2*) or T-DNA insertions (*soc1-2, soc1-6, agl24-3, agl24-4*). Rectangles correspond to exons while lines represent introns. (B) Relative expression levels of *AGL24* in WT, *agl24-4* and *agl24-3* plants and relative expression levels of SOC1 in WT, *soc1-2* and *soc1-6* plants. (C) Relative transcript accumulation levels of *AGL24*, *SOC1* and *XAL2* in WT, *agl24-3, soc1-2, xal2-2 agl24-3* and *xal2-2 soc1-2* plants. (D) Relative expression levels of *AGL24*, *SOC1*, and *XAL2* in WT, *35S::AGL24*, *soc1-101D*, and the double overexpression line *35S::AGL24 soc1-101D* plants. The data represents the mean ± SE of two (for panel B) or three (for panel C and D) independent biological replicates, each with two technical replicates, using roots from 7 dps plants. In both cases, asterisks indicate significant differences compared to WT plants (Wilcoxon test, * P<0.05, *** P<0.0005). We employed *RNAH*, *PDF2*, and *UPL7* as reference genes.


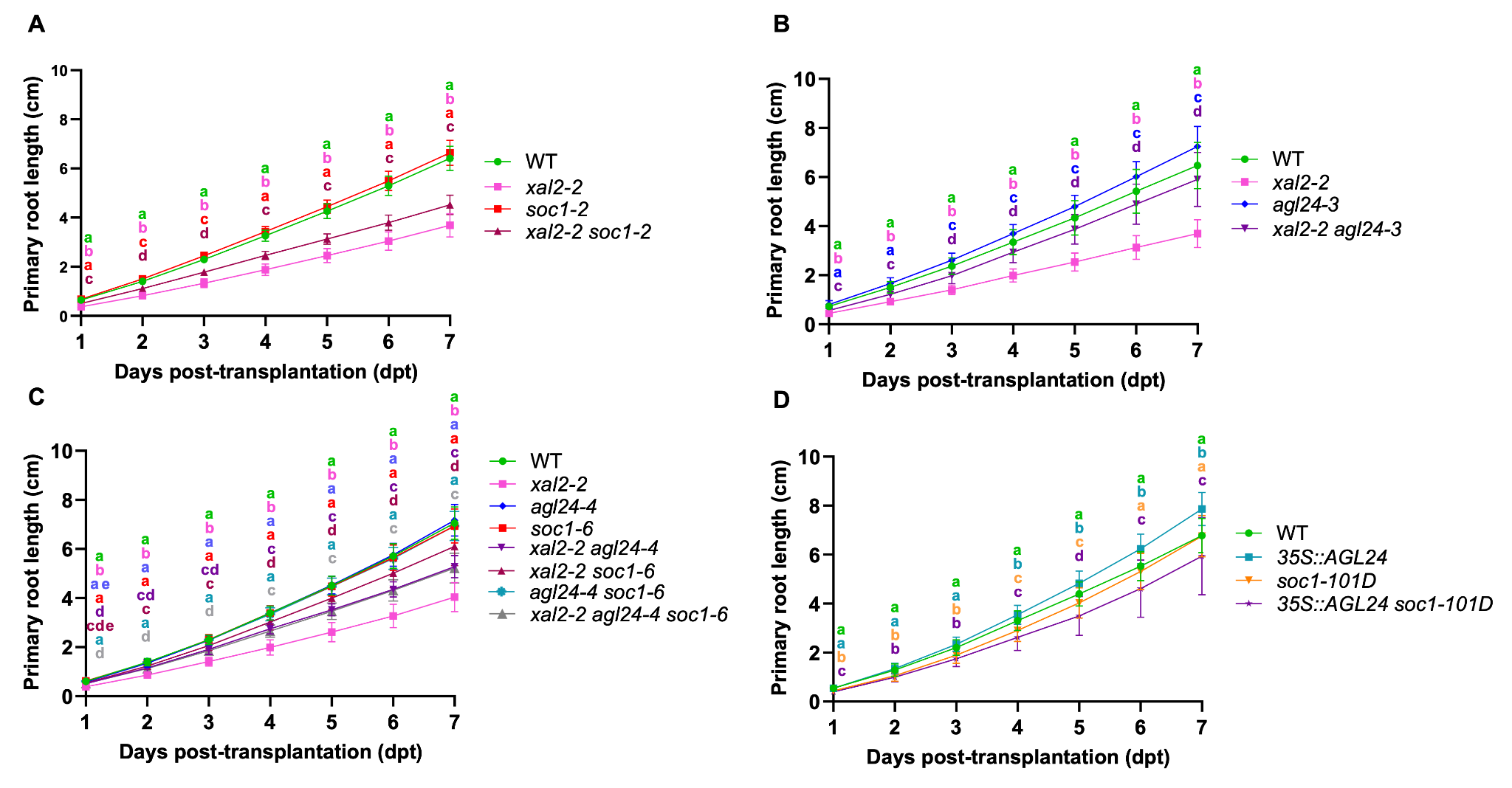


Supplementary Figure 6. **Primary root growth kinetics of the different lines used in this study**. Primary root length (cm) of (A) WT, *xal2-2, soc1-2* and the double mutant *xal2-2 soc1-2* (n ≥ 85), (B) WT, *xal2-2, agl24-3* and the double mutant *xal2-2 agl24-3* (n≥ 75), (C) WT, *xal2-2*, *agl24-4*, *soc1-6*, the double and triple mutants *xal2-2 agl24-4, xal2-2 soc1-6, agl24-4 soc1-6,* and *xal2-2 agl24-4 soc1-6* (n ≥ 85) and (D) WT, *35S::AGL24, soc1-101D* and the double overexpression line *35S::AGL24 soc1-101D* (n ≥ 85). In all cases, data are presented as the mean of three independent biological replicates. The different mutant and overexpression lines are represented with different colors and the different letters indicate significant differences as determined by Kruskal-Wallis test followed by Dunn’s *post-hoc* test.


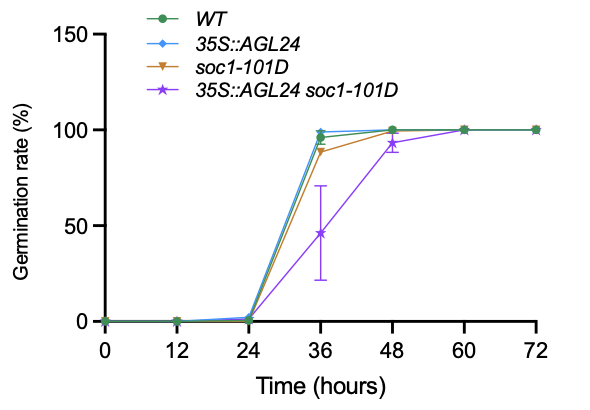


Supplementary Figure 7**. Germination rate (%) in WT, *35S::AGL24*, *soc1-101D* and *35S::AGL24 soc1-101D* seeds**. Germination of WT (n=179), *35S::AGL24* (n=181), *soc1-101D* (n=181) and the double overexpression line *35S::AGL24 soc1-101D* (n=150) seeds with measurements for 0, 12, 24, 36, 48, 60 and 72 hours after sowing.


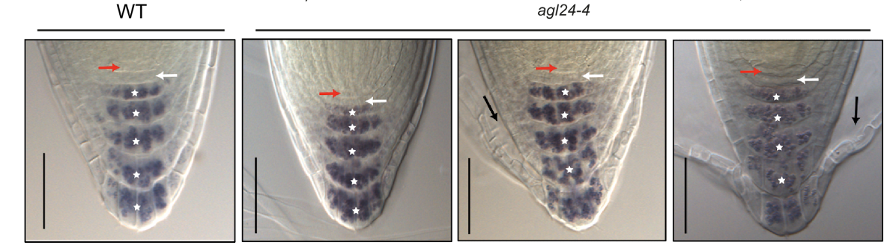


Supplementary Figure 8. **Representative images of *agl24-4* used as an example for the counting of the DCCs**. Representative images of the root tip of WT and *agl24-4* plants showing the QC with a red arrow, the columella stem cell layer in white arrow and the differentiated columella cell layers with an asterisk. The black arrows show the last differentiated columella layer that is partially detached from the root, which was not counted as a layer. Bars, 50 μM.


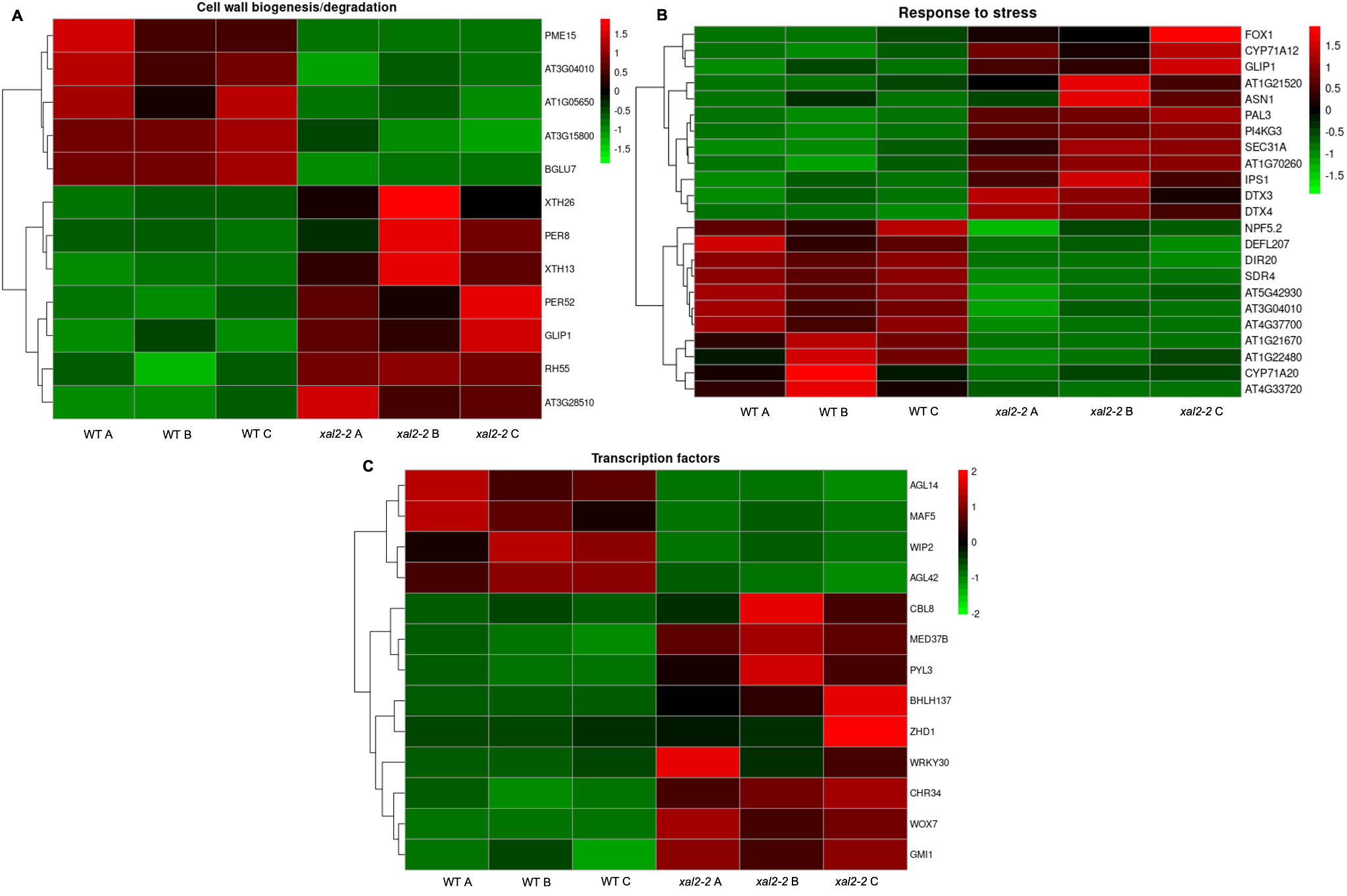


Supplementary Figure 9. **Heatmaps of selected DEGs from different GO categories: (A) Water stress, (B) Cell wall organization and biogenesis and (C) Transcription factors.** Three biological replicates of RNA-seq (labeled as A, B and C in each case) were performed on 7 dps WT and *xal2-2* roots. Red and green in heatmap show unigenes with higher and lower expression levels, respectively. Color changes indicate changes in gene expression levels, respectively.

**Supplementary Table 1.** Multiple comparisons of root lengths among the different genetic backgrounds at 7 dpt. Results for Kruskal-Wallis test followed by Dunn’s *post-hoc* test. ns= not significant; * p< 0.033; ** p< 0.0021; *** p< 0.0002; **** p < 0.0001.

| **Dunn's multiple comparisons test** | **Mean rank diff.** | **Significant?** | **Summary** | **Adjusted P Value** |
| --- | --- | --- | --- | --- |
| WT vs. *xal2-2* | 731.5 | Yes | **** | <0.0001 |
| WT vs. *agl24-4* | -42.94 | No | ns | >0.9999 |
| WT vs. *soc1-6* | 17.58 | No | ns | >0.9999 |
| WT vs. *xal2-2 agl24-4* | 520.8 | Yes | **** | <0.0001 |
| WT vs. *xal2-2 soc1-6* | 284.8 | Yes | **** | <0.0001 |
| WT vs. *agl24-4 soc1-6* | -1.548 | No | ns | >0.9999 |
| WT vs. *xal2-2 agl24-4 soc1-6* | 546.2 | Yes | **** | <0.0001 |
| WT vs. *35S::AGL24* | -231.9 | Yes | *** | 0.0001 |
| WT vs. *soc1-101D* | 101.7 | No | ns | >0.9999 |
| WT vs. *35S::AGL24 soc1-101D* | 276.6 | Yes | **** | <0.0001 |
| *xal2-2 vs. agl24-4* | -774.5 | Yes | **** | <0.0001 |
| *xal2-2 vs. soc1-6* | -714.0 | Yes | **** | <0.0001 |
| *xal2-2 vs. xal2-2 agl24-4* | -210.7 | Yes | *** | 0.0002 |
| *xal2-2 vs. xal2-2 soc1-6* | -446.7 | Yes | **** | <0.0001 |
| *xal2-2 vs. agl24-4 soc1-6* | -733.1 | Yes | **** | <0.0001 |
| *xal2-2 vs. xal2-2 agl24-4 soc1-6* | -185.3 | Yes | ** | 0.0028 |
| *xal2-2 vs. 35S::AGL24* | -963.4 | Yes | **** | <0.0001 |
| *xal2-2 vs. soc1-101D* | -629.9 | Yes | **** | <0.0001 |
| *xal2-2 vs. 35S::AGL24 soc1-101D* | -454.9 | Yes | **** | <0.0001 |
| *agl24-4 vs. soc1-6* | 60.52 | No | ns | >0.9999 |
| *agl24-4 vs. xal2-2 agl24-4* | 563.8 | Yes | **** | <0.0001 |
| *agl24-4 vs. xal2-2 soc1-6* | 327.7 | Yes | **** | <0.0001 |
| *agl24-4 vs. agl24-4 soc1-6* | 41.39 | No | ns | >0.9999 |
| *agl24-4 vs. xal2-2 agl24-4 soc1-6* | 589.2 | Yes | **** | <0.0001 |
| *agl24-4 vs. 35S::AGL24* | -188.9 | Yes | ** | 0.0071 |
| *agl24-4 vs. soc1-101D* | 144.6 | No | ns | 0.1914 |
| *agl24-4 vs. 35S::AGL24 soc1-101D* | 319.6 | Yes | **** | <0.0001 |
| *soc1-6 vs. xal2-2 agl24-4* | 503.3 | Yes | **** | <0.0001 |
| *soc1-6 vs. xal2-2 soc1-6* | 267.2 | Yes | **** | <0.0001 |
| *soc1-6 vs. agl24-4 soc1-6* | -19.13 | No | ns | >0.9999 |
| *soc1-6 vs. xal2-2 agl24-4 soc1-6* | 528.6 | Yes | **** | <0.0001 |
| *soc1-6 vs. 35S::AGL24* | -249.4 | Yes | **** | <0.0001 |
| *soc1-6 vs. soc1-101D* | 84.08 | No | ns | >0.9999 |
| *soc1-6 vs. 35S::AGL24 soc1-101D* | 259.0 | Yes | **** | <0.0001 |
| *xal2-2 agl24-4 vs. xal2-2 soc1-6* | -236.0 | Yes | **** | <0.0001 |
| *xal2-2 agl24-4 vs. agl24-4 soc1-6* | -522.4 | Yes | **** | <0.0001 |
| *xal2-2 agl24-4 vs. xal2-2 agl24-4 soc1-6* | 25.38 | No | ns | >0.9999 |
| *xal2-2 agl24-4 vs. 35S::AGL24* | -752.7 | Yes | **** | <0.0001 |
| *xal2-2 agl24-4 vs. soc1-101D* | -419.2 | Yes | **** | <0.0001 |
| *xal2-2 agl24-4 vs. 35S::AGL24 soc1-101D* | -244.2 | Yes | **** | <0.0001 |
| *xal2-2 soc1-6 vs. agl24-4 soc1-6* | -286.4 | Yes | **** | <0.0001 |
| *xal2-2 soc1-6 vs. xal2-2 agl24-4 soc1-6* | 261.4 | Yes | **** | <0.0001 |
| *xal2-2 soc1-6 vs. 35S::AGL24* | -516.7 | Yes | **** | <0.0001 |
| *xal2-2 soc1-6 vs. soc1-101D* | -183.1 | Yes | * | 0.0121 |
| *xal2-2 soc1-6 vs. 35S::AGL24 soc1-101D* | -8.178 | No | ns | >0.9999 |
| *agl24-4 soc1-6 vs. xal2-2 agl24-4 soc1-6* | 547.8 | Yes | **** | <0.0001 |
| *agl24-4 soc1-6 vs. 35S::AGL24* | -230.3 | Yes | *** | 0.0002 |
| *agl24-4 soc1-6 vs. soc1-101D* | 103.2 | No | ns | >0.9999 |
| *agl24-4 soc1-6 vs. 35S::AGL24 soc1-101D* | 278.2 | Yes | **** | <0.0001 |
| *xal2-2 agl24-4 soc1-6 vs. 35S::AGL24* | -778.1 | Yes | **** | <0.0001 |
| *xal2-2 agl24-4 soc1-6 vs. soc1-101D* | -444.6 | Yes | **** | <0.0001 |
| *xal2-2 agl24-4 soc1-6 vs. 35S::AGL24 soc1-101D* | -269.6 | Yes | **** | <0.0001 |
| *35S::AGL24 vs. soc1-101D* | 333.5 | Yes | **** | <0.0001 |
| *35S::AGL24 vs. 35S::AGL24 soc1-101D* | 508.5 | Yes | **** | <0.0001 |
| *soc1-101D vs. 35S::AGL24 soc1-101D* | 175.0 | No | ns | 0.0516 |

**Supplementary Table 2.** Percentage of CSC and DCC layers in the different genetic backgrounds used in this work.

| **% of CSCs layers** | ***WT*** | ***xal2-***  ***2*** | ***agl24-4*** | ***soc1-6*** | ***xal2-2 agl24-4*** | ***xal2-2 soc1-6*** | ***agl24-4 soc1-6*** | ***xal2-2 agl24-4 soc1-6*** | ***35S:***  ***AGL24*** | ***soc1-101D*** | ***35S:AGL24 soc1-101D 5d*** | ***35S:AGL24 soc1-101D 6d*** |
| --- | --- | --- | --- | --- | --- | --- | --- | --- | --- | --- | --- | --- |
| **0** | 22.53 | 25.21 | 21.11 | 31.11 | 35.56 | 34.44 | 16.67 | 41.11 | 11.56 | 1.25 | 2.67 | 3.33 |
| **1** | 62.95 | 59.00 | 64.44 | 46.67 | 56.67 | 56.67 | 67.78 | 45.56 | 63.44 | 55.07 | 57.99 | 77.22 |
| **2** | 14.52 | 15.79 | 14.44 | 22.22 | 7.78 | 8.89 | 15.56 | 13.33 | 25.00 | 43.68 | 39.34 | 19.44 |
| **% of DCCs layers** | ***WT*** | ***xal2-2*** | ***agl24-4*** | ***soc1-6*** | ***xal2-2 agl24-4*** | ***xal2-2 soc1-6*** | ***agl24-4 soc1-6*** | ***xal2-2 agl24-4 soc1-6*** | ***35S:***  ***AGL24*** | ***soc1-101D*** | ***35S:AGL24 soc1-101D 5d*** | ***35S:AGL24 soc1-101D 6d*** |
| **3** | 0.00 | 0.00 | 0.00 | 0.00 | 0.00 | 0.00 | 0.00 | 0.00 | 0.00 | 8.33 | 22.65 | 2.78 |
| **4** | 12.34 | 24.83 | 36.67 | 25.56 | 14.44 | 20.00 | 15.56 | 13.33 | 31.77 | 67.76 | 61.88 | 60.56 |
| **5** | 73.03 | 51.11 | 52.22 | 54.44 | 55.56 | 56.67 | 73.33 | 57.78 | 58.75 | 21.65 | 15.47 | 33.89 |
| **6** | 14.64 | 24.06 | 11.11 | 20.00 | 30.00 | 23.33 | 11.11 | 28.89 | 9.48 | 2.25 | 0.00 | 2.78 |

**Supplementary Table 3.** Multiple comparisons of CSC and DCC layers among the different genetic backgrounds analyzed in this study. Results for Fisher test followed by Bonferroni correction for multiple comparisons. * p< 0.05; ** p< 0.01; *** p< 0.0001.

**Proportion of columella stem cell layers**

| **Comparison (columella stem cell layers)** | **p.Fisher** | **p.adj.Fisher** | **Significant?** |
| --- | --- | --- | --- |
| WT vs *xal2-2* | 0.88714 | 0.91486 |  |
| WT vs *agl24-4* | 0.9756 | 0.99061 |  |
| WT vs *soc1-6* | 0.087952 | 0.135 |  |
| WT vs *xal2-2_agl24-4* | 0.092936 | 0.1394 |  |
| WT vs *xal2-2_soc1-6* | 0.15259 | 0.20981 |  |
| WT vs *agl24-4_soc1-6* | 0.66709 | 0.72177 |  |
| WT vs *xal2-2_agl24-4_soc1-6* | 0.025163 | 0.044885 | * |
| WT vs *35S::AGL24* | 0.19624 | 0.25904 |  |
| WT vs *soc1-101D* | 9.989e-9 | 7.3253e-8 | *** |
| WT vs *35S::AGL24 soc1-101D 5d* | 1.9732e-6 | 9.3022e-6 | *** |
| WT vs *35S::AGL24 soc1-101D 6d* | 0.012659 | 0.026951 | * |
| *xal2-2 vs agl24-4* | 0.75048 | 0.7989 |  |
| *xal2-2 vs soc1-6* | 0.24368 | 0.30345 |  |
| *xal2-2 vs xal2-2_agl24-4* | 0.13479 | 0.18928 |  |
| *xal2-2 vs xal2-2_soc1-6* | 0.21635 | 0.2746 |  |
| *xal2-2 vs agl24-4_soc1-6* | 0.34738 | 0.39529 |  |
| *xal2-2 vs xal2-2_agl24-4_soc1-6* | 0.072575 | 0.11405 |  |
| *xal2-2 vs 35S::AGL24* | 0.12571 | 0.18037 |  |
| *xal2-2 vs soc1-101D* | 6.0643e-9 | 5.003e-8 | *** |
| *xal2-2 vs 35S::AGL24 soc1-101D 5d* | 1.077e-6 | 5.4678e-6 | *** |
| *xal2-2 vs 35S::AGL24 soc1-101D 6d* | 0.0057138 | 0.013967 | * |
| *agl24-4 vs soc1-6* | 0.059554 | 0.10078 |  |
| *agl24-4 vs xal2-2_agl24-4* | 0.069898 | 0.11252 |  |
| *agl24-4 vs xal2-2_soc1-6* | 0.10524 | 0.15435 |  |
| *agl24-4 vs agl24-4_soc1-6* | 0.77567 | 0.81261 |  |
| *agl24-4 vs xal2-2_agl24-4_soc1-6* | 0.010778 | 0.023712 | * |
| *agl24-4 vs 35S::AGL24* | 0.25022 | 0.30582 |  |
| *agl24-4 vs soc1-101D* | 1.7031e-8 | 1.124e-7 | *** |
| *agl24-4 vs 35S::AGL24 soc1-101D 5d* | 3.4446e-6 | 1.4209e-5 | *** |
| *agl24-4 vs 35S::AGL24 soc1-101D 6d* | 0.014838 | 0.029676 | * |
| *soc1-6 vs xal2-2_agl24-4* | 0.024591 | 0.044885 | * |
| *soc1-6 vs xal2-2_soc1-6* | 0.049679 | 0.086285 |  |
| *soc1-6 vs agl24-4_soc1-6* | 0.014259 | 0.029409 | * |
| *soc1-6 vs xal2-2_agl24-4_soc1-6* | 0.2029 | 0.26258 |  |
| *soc1-6 vs 35S::AGL24* | 0.0090771 | 0.021396 | * |
| *soc1-6 vs soc1-101D* | 1.1199e-9 | 1.0559e-8 | *** |
| *soc1-6 vs 35S::AGL24 soc1-101D 5d* | 1.4925e-7 | 8.955e-7 | *** |
| *soc1-6 vs 35S::AGL24 soc1-101D 6d* | 3.1222e-4 | 9.3666e-4 | *** |
| *xal2-2_agl24-4 vs xal2-2_soc1-6* | 1 | 1 |  |
| *xal2-2_agl24-4 vs agl24-4_soc1-6* | 0.0099083 | 0.02255 | * |
| *xal2-2_agl24-4 vs xal2-2_agl24-4_soc1-6* | 0.28119 | 0.33743 |  |
| *xal2-2_agl24-4 vs 35S::AGL24* | 4.6949e-4 | 0.0013472 | ** |
| *xal2-2_agl24-4 vs soc1-101D* | 9.4394e-15 | 6.23e-13 | *** |
| *xal2-2_agl24-4 vs 35S::AGL24 soc1-101D 5d* | 5.6429e-12 | 8.8666e-11 | *** |
| *xal2-2_agl24-4 vs 35S::AGL24 soc1-101D 6d* | 1.1945e-5 | 4.6375e-5 | *** |
| *xal2-2_soc1-6 vs agl24-4_soc1-6* | 0.015621 | 0.030323 | * |
| *xal2-2_soc1-6 vs xal2-2_agl24-4_soc1-6* | 0.2969 | 0.34378 |  |
| *xal2-2_soc1-6 vs 35S::AGL24* | 0.0011373 | 0.0030025 | ** |
| *xal2-2_soc1-6 vs soc1-101D* | 5.4019e-14 | 1.1884e-12 | *** |
| *xal2-2_soc1-6 vs 35S::AGL24 soc1-101D 5d* | 3.6385e-11 | 4.0024e-10 | *** |
| *xal2-2_soc1-6 vs 35S::AGL24 soc1-101D 6d* | 3.2588e-5 | 1.1949e-4 | *** |
| *agl24-4_soc1-6 vs xal2-2_agl24-4_soc1-6* | 0.0010865 | 0.0029879 | ** |
| *agl24-4_soc1-6 vs 35S::AGL24* | 0.5586 | 0.61446 |  |
| *agl24-4_soc1-6 vs soc1-101D* | 3.3757e-7 | 1.8566e-6 | *** |
| *agl24-4_soc1-6 vs 35S::AGL24 soc1-101D 5d* | 5.9798e-5 | 2.0772e-4 | *** |
| *agl24-4_soc1-6 vs 35S::AGL24 soc1-101D 6d* | 0.067921 | 0.11207 |  |
| *xal2-2_agl24-4_soc1-6 vs 35S::AGL24* | 1.3751e-4 | 4.3217e-4 | *** |
| *xal2-2_agl24-4_soc1-6 vs soc1101D* | 1.9797e-14 | 6.533e-13 | *** |
| *xal2-2_agl24-4_soc1-6 vs 35S::AGL24 soc1-101D 5d* | 6.7171e-12 | 8.8666e-11 | *** |
| *xal2-2_agl24-4_soc1-6 vs 35S::AGL24 soc1-101D 6d* | 3.4376e-6 | 1.4209e-5 | *** |
| *35S::AGL24 vs soc1-101D* | 8.6532e-5 | 2.8556e-4 | *** |
| *35S::AGL24 vs 35S::AGL24 soc1-101D 5d* | 0.0043603 | 0.011068 | * |
| *35S::AGL24 vs 35S::AGL24 soc1-101D 6d* | 0.29572 | 0.34378 |  |
| *soc1101D vs 35S::AGL24 soc1-101D 5d* | 0.47893 | 0.53575 |  |
| *soc1101D vs 35S::AGL24 soc1-101D 6d* | 0.024163 | 0.044885 | * |
| *35S::AGL24 soc1-101D 5d vs 35S::AGL24 soc1-101D 6d* | 0.17378 | 0.23407 |  |

**Supplementary Table 3 (continued)**

**Proportion of differentiated columella cell layers**

| **Comparison (differentiated columella cell layers)** | **p.Fisher** | **p.adj.Fisher** | **Significant?** |
| --- | --- | --- | --- |
| WT vs *xal2-2* | 0.010683 | 0.019586 | * |
| WT vs *agl24-4* | 7.4781e-4 | 0.0017019 | ** |
| WT vs *soc1-6* | 0.026274 | 0.044464 | * |
| WT vs *xal2-2_agl24-4* | 0.029613 | 0.048861 | * |
| WT vs *xal2-2_soc1-6* | 0.075565 | 0.1063 |  |
| WT vs *agl24-4_soc1-6* | 0.69289 | 0.71454 |  |
| WT vs *xal2-2_agl24-4_soc1-6* | 0.056992 | 0.083588 |  |
| WT vs *35S::AGL24* | 0.075699 | 0.1063 |  |
| WT vs *soc1-101D* | 1.5725e-18 | 1.7298e-17 | *** |
| WT vs *35S::AGL24 soc1-101D* 5d | 1.47069e-21 | 4.8533e-20 | *** |
| WT vs *35S::AGL24 soc1-101D* 6d | 6.1315e-10 | 2.1299e-9 | *** |
| *xal2-2 vs agl24-4* | 0.048532 | 0.072798 |  |
| *xal2-2 vs soc1-6* | 0.83028 | 0.84305 |  |
| *xal2-2 vs xal2-2_agl24-4* | 0.19739 | 0.24125 |  |
| *xal2-2 vs xal2-2_soc1-6* | 0.68653 | 0.71454 |  |
| *xal2-2 vs agl24-4_soc1-6* | 0.007519 | 0.014179 | * |
| *xal2-2 vs xal2-2_agl24-4_soc1-6* | 0.136 | 0.1703 |  |
| *xal2-2 vs 35S::AGL24* | 0.10432 | 0.13827 |  |
| *xal2-2 vs soc1-101D* | 2.0041e-12 | 9.4479e-12 | *** |
| *xal2-2 vs 35S::AGL24 soc1-101D 5d* | 3.2161e-15 | 2.1226e-14 | *** |
| *xal2-2 vs 35S::AGL24 soc1-101D 6d* | 2.2358e-6 | 6.1484e-6 | *** |
| *agl24-4 vs soc1-6* | 0.13676 | 0.1703 |  |
| *agl24-4 vs xal2-2_agl24-4* | 2.3172e-4 | 5.462e-4 | *** |
| *agl24-4 vs xal2-2_soc1-6* | 0.014522 | 0.025222 | * |
| *agl24-4 vs agl24-4_soc1-6* | 0.0040836 | 0.0086941 | ** |
| *agl24-4 vs xal2-2_agl24-4_soc1-6* | 1.5636e-4 | 3.8221e-4 | *** |
| *agl24-4 vs 35S::AGL24* | 0.30683 | 0.36162 |  |
| *agl24-4 vs soc1-101D* | 2.232e-7 | 6.4049e-7 | *** |
| *agl24-4 vs 35S::AGL24 soc1-101D 5d* | 7.0333e-10 | 2.321e-9 | *** |
| *agl24-4 vs 35S::AGL24 soc1-101D 6d* | 0.0027333 | 0.0060133 | ** |
| *soc1-6 vs xal2-2_agl24-4* | 0.10475 | 0.13827 |  |
| *soc1-6 vs xal2-2_soc1-6* | 0.65772 | 0.70015 |  |
| *soc1-6 vs agl24-4_soc1-6* | 0.030978 | 0.049867 | * |
| *soc1-6 vs xal2-2_agl24-4_soc1-6* | 0.081803 | 0.11248 |  |
| *soc1-6 vs 35S::AGL24* | 0.31271 | 0.36209 |  |
| *soc1-6 vs soc1-101D* | 4.8452e-12 | 2.1319e-11 | *** |
| *soc1-6 vs 35S::AGL24 soc1-101D 5d* | 1.0005e-14 | 5.5028e-14 | *** |
| *soc1-6 vs 35S::AGL24 soc1-101D 6d* | 5.9935e-6 | 1.5823e-5 | *** |
| *xal2-2_agl24-4 vs xal2-2_soc1-6* | 0.47487 | 0.51379 |  |
| *xal2-2_agl24-4 vs agl24-4_soc1-6* | 0.0060277 | 0.012127 | * |
| *xal2-2_agl24-4 vs xal2-2_agl24-4_soc1-6* | 0.95555 | 0.95555 |  |
| *xal2-2_agl24-4 vs 35S::AGL24* | 0.0060633 | 0.012127 | * |
| *xal2-2_agl24-4 vs soc1-101D* | 2.2199e-18 | 2.093e-17 | *** |
| *xal2-2_agl24-4 vs 35S::AGL24 soc1-101D 5d* | 3.1345999999999998e-21 | 6.8961e-20 | *** |
| *xal2-2_agl24-4 vs 35S::AGL24 soc1-101D 6d* | 5.9743e-10 | 2.1299e-9 | *** |
| *xal2-2_soc1-6 vs agl24-4_soc1-6* | 0.042044 | 0.066069 |  |
| *xal2-2_soc1-6 vs xal2-2_agl24-4_soc1-6* | 0.41243 | 0.45367 |  |
| *xal2-2_soc1-6 vs 35S::AGL24* | 0.1237 | 0.16008 |  |
| *xal2-2_soc1-6 vs soc1-101D* | 8.9881e-15 | 5.3929e-14 | *** |
| *xal2-2_soc1-6 vs 35S::AGL24 soc1-101D 5d* | 1.1423e-17 | 9.424e-17 | *** |
| *xal2-2_soc1-6 vs 35S::AGL24 soc1-101D 6d* | 1.2045e-7 | 3.6135e-7 | *** |
| *agl24-4_soc1-6 vs xal2-2_agl24-4_soc1-6* | 0.011935 | 0.021289 | * |
| *agl24-4_soc1-6 vs 35S::AGL24* | 0.22147 | 0.26576 |  |
| *agl24-4_soc1-6 vs soc1-101D* | 1.0724e-16 | 7.8643e-16 | *** |
| *agl24-4_soc1-6 vs 35S::AGL24 soc1-101D 5d* | 2.5347e-19 | 4.1823e-18 | *** |
| *agl24-4_soc1-6 vs 35S::AGL24 soc1-101D 6d* | 1.086e-8 | 3.4131e-8 | *** |
| *xal2-2_agl24-4_soc1-6 vs 35S::AGL24* | 0.0070179 | 0.013623 | * |
| *xal2-2_agl24-4_soc1-6 vs soc1-101D* | 6.3399e-19 | 8.3687e-18 | *** |
| *xal2-2_agl24-4_soc1-6 vs 35S::AGL24 soc1-101D 5d* | 6.5066e-22 | 4.2944000000000004e-20 | *** |
| *xal2-2_agl24-4_soc1-6 vs 35S::AGL24 soc1-101D 6d* | 2.6463e-10 | 1.0274e-9 | *** |
| *35S::AGL24 vs soc1-101D* | 4.0015e-11 | 1.6506e-10 | *** |
| *35S::AGL24 vs 35S::AGL24 soc1-101D 5d* | 9.1899e-14 | 4.6656e-13 | *** |
| *35S::AGL24 vs 35S::AGL24 soc1-101D 6d* | 2.8409e-5 | 7.2115e-5 | *** |
| *soc1-101D vs 35S::AGL24 soc1-101D 5d* | 0.40047 | 0.45367 |  |
| *soc1-101D vs 35S::AGL24 soc1-101D 6d* | 0.40984 | 0.45367 |  |
| *35S::AGL24 soc1-101D 5d vs 35S::AGL24 soc1-101D 6d* | 0.047196 | 0.07244 |  |

**Supplementary Table 4:** List of 20 most differentially expressed genes found in the data from the RNA-seq when comparing *xal2-2* vs WT.

| **Gene ID** | **log2Fold**  **Change** | **padj** | **Gen name** | **Gen description** |
| --- | --- | --- | --- | --- |
| AT5G24240 | 4.16 | 2.65E-134 | *PI4KG3* | Phosphatidylinositol 4-kinase gamma 3 |
| AT1G55210 | -3.19 | 3.4493E-100 | *DIR20* | Dirigent protein 20 |
| AT3G29250 | -1.89 | 2.25555E-41 | *SDR4* | Short-chain dehydrogenase reductase 4 |
| AT1G21670 | -3.26 | 2.70274E-38 | - | DPP6 amino-terminal domain protein |
| AT4G11880 | -3.48 | 1.14522E-31 | *XAL2/AGL14* | MADS-domain protein AGAMOUS-LIKE AGL14 |
| AT1G77690 | -1.61 | 7.59479E-31 | *LAX3* | Encodes an auxin influx carrier LAX3 (Like Aux1) that promotes lateral root emergence. |
| AT5G23840 | -1.59 | 3.86353E-20 | - | MD-2-related lipid recognition domain-containing protein |
| AT5G60250 | 3.77 | 8.05794E-17 | - | zinc finger (C3HC4-type RING finger) family protein |
| AT3G04010 | -1.64 | 8.05794E-17 | - | O-Glycosyl hydrolases family 17 protein |
| AT5G56080 | 1.60 | 2.59056E-14 | *NAS2* | NICOTIANAMINE SYNTHASE 2 |
| AT4G05275 | 10.16 | 9.06755E-14 | - | long_noncoding_rna |
| AT2G30750 | 1.87 | 5.55305E-13 | *CYP71A12* | CYTOCHROME P450, FAMILY 71, SUBFAMILY A |
| AT3G60160 | 3.14 | 1.18622E-11 | *ABCC9* | ATP-BINDING CASSETTE C9 |
| AT1G75945 | -9.51 | 1.92412E-11 | - | hypothetical protein |
| AT4G04990 | -2.06 | 7.51825E-11 | *DUF761* | serine/arginine repetitive matrix-like protein |
| AT4G33720 | -3.05 | 7.51825E-11 | *ATCAPE3* | CAP (Cysteine-rich secretory proteins, Antigen 5, and Pathogenesis-related 1 protein) superfamily protein |
| AT5G62165 | -1.55 | 1.02229E-10 | *AGL42* | MADS-domain protein AGAMOUS-LIKE AGL42 |
| AT4G37700 | -1.50 | 1.44253E-10 | - | hypothetical protein |
| AT1G05650 | -1.65 | 2.72841E-10 | - | Pectin lyase-like superfamily protein |
| AT2G01020 | 1.73 | 3.17145E-10 | 5.8SrRNA | Ribosomal RNA |
| AT5G57540 | 1.54 | 2.55938E-09 | *XTH13* | XYLOGLUCAN ENDOTRANSGLUCOSYLASE/ HYDROLASE 13 |

**Supplementary Table 5.** Primers used in the RT-qPCR amplifications to determine the relative expression of *XAL2*, *SOC1*, *AGL24*, and the housekeeping references genes *RNAH*, *PDF2* and *UPL7*.

| Gene | Primer name | Direction | Primer sequence |
| --- | --- | --- | --- |
| *RNAH* | RNAH_Y_qF | forward | 5´-CCATTCTACTTTTTGGCGGCT-3´ |
|  | RNAH_Y_qR | reverse | 5´-TCAATGGTAACTGATCCACTCTGATG-3´ |
| *PDF2* | PDF2-F1_TR_RC | forward | 5´-TAACGTGGCCAAAATGATGC-3´ |
|  | PDF2-R1_TR_RC | reverse | 5´-GTTCTCCACAACCGCTTGGT-3´ |
| *UPL7* | UPL7-F1_TR_RC | forward | 5´-TTCAAATACTTGCAGCCAACCTT-3´ |
|  | UPL7-R1_TR_RC | reverse | 5´-CCCAAAGAGAGGTATCACAAGAGACT-3´ |
| *XAL2* | XAL2-Q-F2 | forward | 5´-GATAATTCACAGCAATCGAAGG-3´ |
|  | XAL2-Q-R2 | reverse | 5´-GGTTCTCCAATTGTTGTAACTC -3´ |
| *SOC1* | SOC1_SQ_qF2 | forward | 5´-CTCTCTGAAAAGTGGGGATC-3´ |
|  | SOC1_SQ_qR2 | reverse | 5´-AGAACTTGGGCTACTCTCTTC-3´ |
| *AGL24* | AGL24-Real-Time-F | forward | 5´-GAGGCTTTGGAGACAGAGTCGGTGA-3´ |
|  | AGL24-Real-time-R | reverse | 5´-AGATGGAAGCCCAAGCTTCAGGGAA-3´ |
| *WOX5* | qPCR_WOX5_F | forward | 5’-CCATCAACTAGAGATGTTTTTG-3’ |
|  | qPCR_WOX5_R | reverse | 5’-CACCTTGGAGTTGGAGTCTTC-3’ |
| *AGL42* | qPCR_AGL42_F | forward | 5’-GAACGCTACCGCAAGTACAC-3’ |
|  | qPCR_AGL42_R | reverse | 5’-GCAAGTGAATTTGTGAGTCGTG-3’ |
| *PER8* | AT1G34510_CC_qF | forward | 5’-TGGGTAGAGTTGTTTTCAACC-3’ |
|  | AT1G34510_CC_qR | reverse | 5’-GAAGCATCACAACCTTTGAC-3’ |
| *PI4KG3* | PI4Kgamma3_qF | forward | 5’-AGAGGAAAAAACATATCATCAGAAG-3’ |
|  | PI4Kgamma3_qR | reverse | 5’-GCTCTTTCAGCAATATCGG-3’ |

**Supplementary Table 6.** Primers used for cloning *SOC1* and *XAL2* genomic regions.

| Gene | Primer name | Direction | Primer sequence |
| --- | --- | --- | --- |
| *SOC1* | pSOC1ND FW | forward | 5´-TTATATGTTATGATTTTGCATGAGC-3´ |
|  | SOC1MA-R | reverse | 5´-CTTTCTTGAAGAACAAGGTAACC-3´ |
|  | pSOC1ND-R | reverse | 5´-ATCTTCTTCTTTAGTTAATTTCC-3´ |
| *XAL2* | AGL14F19A (AscI) | forward | 5´-GGCGCGCCGAATGGTGAGGGGAAAGACAGAG-3´ |
|  | OAGR2 | reverse | 5´-GTTTGAAGGAGGAAACTTTTTGAAG-3´ |
|  | AGL14F5P | forward | 5´-CTTGTCCGAGAGGAGCTG-3´ |
|  | PX2-HDNA-RV | reverse | 5´-CATCTCTGTCTTTCCCCTCACCAT-3´ |
| AGL24 | AGL24MA-F | forward | 5´-TTCATGAGACGGAGGGTTCTG-3´ |
|  | AGL24MA-R: | reverse | 5´-CATTTTACCAGATCTCTCCTTCAC-3´ |

**Supplementary Table 7.** Processed dataset of the DEGs found in the RNA-seq (*xal2-2* mutant vs WT). (Separate Excel File).

**Supplementary Table 8.** Complete data measurements of the primary root growth kinetics in the different lines. (Separate Excel File).
